# Baduanjin exercise improves functional capacity and cardiovascular function in older adults with cardiovascular diseases: a systematic review and meta-analysis

**DOI:** 10.3389/fcvm.2025.1419095

**Published:** 2025-04-24

**Authors:** Xingyu Hu, Yiran Li, Haiyun Jiang, Zhihao Luo, Zengqing, Jihua Zou, Xiaoyan Zheng

**Affiliations:** ^1^ School of Rehabilitation Sciences, Southern Medical University, Guangzhou, China; ^2^ School of Laboratory Medicine and Biotechnology, Southern Medical University, Guangzhou, China; ^3^ Quzhou Hospital of TCM at the Junction of Four Provinces Affiliated to Zhejiang Chinese Medical University, Quzhou, China; ^4^ South China Research Center for Acupuncture and Moxibustion, Medical College of Acu-Moxi and Rehabilitation, Guangzhou University of Chinese Medicine, Guangzhou, China; ^5^ Department of Rehabilitation Medicine, Zhujiang Hospital, Southern Medical University, Guangzhou, China

**Keywords:** Baduanjin exercise, functional capacity, cardiovascular function, older adults, meta-analysis

## Abstract

**Background:**

Based on clinical observations and expert recommendations, Baduanjin exercise has demonstrated a positive impact on maintaining and enhancing cardiovascular function. Despite clinical observations of Baduanjin's cardiovascular benefits, systematic evidence for older adults remains limited. Moreover, the effects of Baduanjin exercise on functional capacity in older adults with cardiovascular diseases have not been adequately investigated. Therefore, we conducted a systematic review and meta-analysis to evaluate the overall efficacy and effectiveness of Baduanjin exercise on functional capacity and cardiovascular function in older adults with cardiovascular diseases.

**Methods:**

We conducted a systematic review and meta-analysis, searching related trials through March 7, 2025, across six databases: PubMed, Web of Science, Cochrane Library, China National Knowledge Infrastructure, Wanfang Data, and China Science Journal Database (VIP). Methodological quality was assessed using the Cochrane Risk of Bias Tool, and a meta-analysis of comparative effects was performed using Review Manager v.5.3 software.

**Results:**

A total of 26 randomized controlled trials involving 2,080 participants were included in this meta-analysis. The results indicate that Baduanjin exercise significantly enhances functional capacity as measured by 6MWT compared to the control group (MD: 41.75, 95% CI: 33.08–50.42, *p* < 0.00001, *I*^2^ = 85%), and improves cardiovascular health markers including LVEF (MD: 5.04, 95% CI: 3.40–6.68, *p* < 0.00001, *I*^2^ = 82%). In dose-subgroup analysis, moderate exercise intensity (30–60 min per time) significantly improved cardiovascular function compared with those of the control group (MD: 56.36, 95% CI: 41.49–71.24, *p* < 0.00001, *I*^2^ = 92%). The secondary outcomes also demonstrated benefits in cardiac remodeling indices, including reductions LVESD (MD: −1. 67, 95% CI: −2.76 to −0.59, *p* = 0.002, *I*^2^ = 95%), LVDD (MD: −2.38, 95% CI: −3.59 to −1.17, *p* = 0.0001, *I*^2^ = 93%). In addition, Baduanjin exercise improved blood biomarkers such as NT-proBNP (MD: −183.83, 95% CI: −309.83 to −57.83, *p* < 0.00001, *I*^2^ = 82%) and NO (MD: 3.54, 95% CI: 1.74–5.34, *p* = 0.0001, *I*^2^ = 0%). What's more, it also decreased MLHFQ (MD: −7.00, 95% CI: −9.54 to −4.45, *p* = 0.0003, *I*^2^ = 79%).

**Conclusions:**

Moderate-intensity Baduanjin (30–60 min/session) significantly improved 6MWT distance and cardiac function parameters by LVEF, LVESD, and LVDD, etc.

**Systematic Review Registration:**

identifier, PROSPERO registration number: (CRD42023477008).

## 1 Introduction

The global population is aging at an unprecedented rate, with the number of older adults (aged ≥60 years) projected to reach 2.1 billion by 2050, up from 962 million in 2017, according to the “World Population Prospects” (1). China, in particular, is experiencing a rapid acceleration in both demographic aging and urbanization processes, with aging being a primary risk factor for cardiovascular diseases. This trend has led to a substantial increase in factors influencing the physical and mental well-being, as well as the quality of life, of the elderly population. To mitigate disease progression and attenuate associated morbidity, it is imperative to implement early preventive measures and targeted interventions during critical transitional phases, such as the onset of aging, self-perceived functional deterioration, and emerging cardiovascular compromise.

Cardiovascular diseases, which affect the heart and blood vessels, are commonly treated with medications such as nitrates, antithrombotic drugs, and statins. However, long-term use of these medications may lead to adverse side effects, including arrhythmia, rapid heartbeat, and headaches (2). Exercise, on the other hand, not only improves the pumping function of the heart and peripheral blood circulation but also alleviates mental stress, thereby enhancing the quality of life for patients with cardiovascular diseases.

Baduanjin (Eight-Section Brocade) exercises, as a form of mild-intensity aerobic exercise, consist of eight symmetrical movements, including arm lifting, head shaking, waist turning, and foot flipping (3). Practicing Baduanjin exercises the cardiovascular system, significantly enhancing cardiovascular baseline capabilities. Furthermore, it is conducive to maintaining psychological well-being, thereby enhancing the overall quality of life (4). Despite the growing number of randomized controlled trials (RCTs) investigating the effects of exercise in this field, there is a lack of a comprehensive review of the current best evidence.

Emerging evidence suggests that Baduanjin exerts cardioprotective effects through multifaceted physiological mechanisms (5, 6). First, its slow stretching and controlled breathing patterns enhance endothelial function by upregulating nitric oxide (NO) synthase activity, thereby improving vasodilation and microcirculation (7). Second, the synchronized movement-respiration coordination modulates autonomic nervous balance, increasing parasympathetic dominance (reflected in elevated heart rate variability) while suppressing sympathetic overactivity, which contributes to blood pressure stabilization and stress hormone (e.g., cortisol) reduction (8). Furthermore, the meditative component during exercise activates prefrontal cortical regions, creating top-down regulation of hypothalamic-pituitary-adrenal axis hyperactivity, a critical pathway for chronic disease management (9).

In our study, we conducted a comprehensive meta-analysis to evaluate the impact of Baduanjin exercises on functional capacity in elderly individuals with cardiovascular disease. This assessment included various cardiovascular markers such as the six-minute walk test (6MWT), left ventricular ejection fraction (LVEF), left ventricular end-systolic diameter (LVESD), and left ventricular end-diastolic diameter (LVDD). Additionally, we examined blood markers, including N-terminal pro-brain natriuretic peptide (NT-proBNP) and serum nitric oxide concentration (NO), as well as other indicators from the Minnesota Life Questionnaire for Patients with Heart Failure (MLHFQ).

## 2 Methods

This systematic review and meta-analysis were conducted in strict accordance with the PRISMA 2020 guidelines (10) to ensure methodological rigor, transparency, and reproducibility. The protocol for this review was prospectively designed, including predefined eligibility criteria, search strategies, and data synthesis methods.

### 2.1 Search strategy

A comprehensive search strategy was implemented across six databases, including PubMed, Web of Science, Cochrane Library, China National Knowledge Infrastructure, Wanfang Data, and China Science Journal Database (VIP), to identify all relevant citations published through March 7, 2025. The search strategies employed a combination of Medical Subject Headings and free-text terms related to Baduanjin exercise, cardiovascular diseases, and randomized clinical trials. The detailed search strategies are provided in Supplementary Methods S1.

### 2.2 Selection and exclusion criteria

All available randomized controlled trials (RCTs) were considered eligible if they met the following inclusion criteria: (1) the study population consisted of older adults diagnosed with one or more cardiovascular diseases; (2) the cardiovascular diseases included, but were not limited to, coronary heart disease, heart failure, hypertension, myocardial infarction, angina, and cardiac dysfunction (defined as left ventricular ejection fraction ≤40%); (3) the intervention involved Baduanjin exercise, with a comparison to usual care control or other therapy; and (4) the study reported at least one outcome measure assessing cardiac performance.

### 2.3 Data extraction and quality assessment

Studies were excluded if they presented insufficient data or irrelevant outcomes. Additionally, conference abstracts, clinical protocols, case reports, review articles, and animal experimental studies were deemed ineligible for inclusion.

Two independent investigators (L.Y.R. and H.X.Y.) extracted data and relevant information from the eligible studies. Basic information about the included studies, encompassing population, intervention, comparison, and outcome, was extracted and summarized in a standardized table. The methodological quality of the included studies was evaluated by two reviewers (L.Y.R. and H.X.Y.) using the Cochrane Risk of Bias Tool (11). Any discrepancies were resolved through consultation with a third reviewer (Z.X.Y.) to reach a consensus.

### 2.4 Outcomes

The primary outcomes were categorized into two domains: (1) Functional exercise capacity, assessed via the 6MWT, which quantifies submaximal aerobic endurance and correlates with activities of daily living performance; and (2) Cardiovascular function, including cardiac structural indices (LVEF, LVESD, LVDD) and blood biomarkers (NT-proBNP, nitric oxide).

### 2.5 Statistical analysis

The meta-analysis was performed using Review Manager v.5.3 software (Cochrane Collaboration, Oxford, UK). Continuous outcomes were analyzed using the mean difference (MD) or standardized mean difference (SMD) with 95% confidence intervals (CIs) was used to analyze continuous outcomes. The SMD statistic was employed when the outcome was assessed using different scales across studies. *I*^2^ statistics were calculated to assess the heterogeneity and to choose the effect model. If *I*^2^ *>* 50% and the *P*-value of the *χ*^2^ was less than 0.1, indicating the presence of significant statistical heterogeneity, a random-effects model was applied. Otherwise, a fixed-effects model was used. In the event of clinical heterogeneity in the pooled results, subgroup analysis was conducted to identify potential sources of heterogeneity. The certainty of evidence was assessed using the GRADE criteria. All methodological details, including search strategies, selection processes, and synthesis protocols, are reported in the PRISMA 2020 Checklist (Supplementary Table S2).

## 3 Results

### 3.1 Study identification and selection

**Figure 1:**
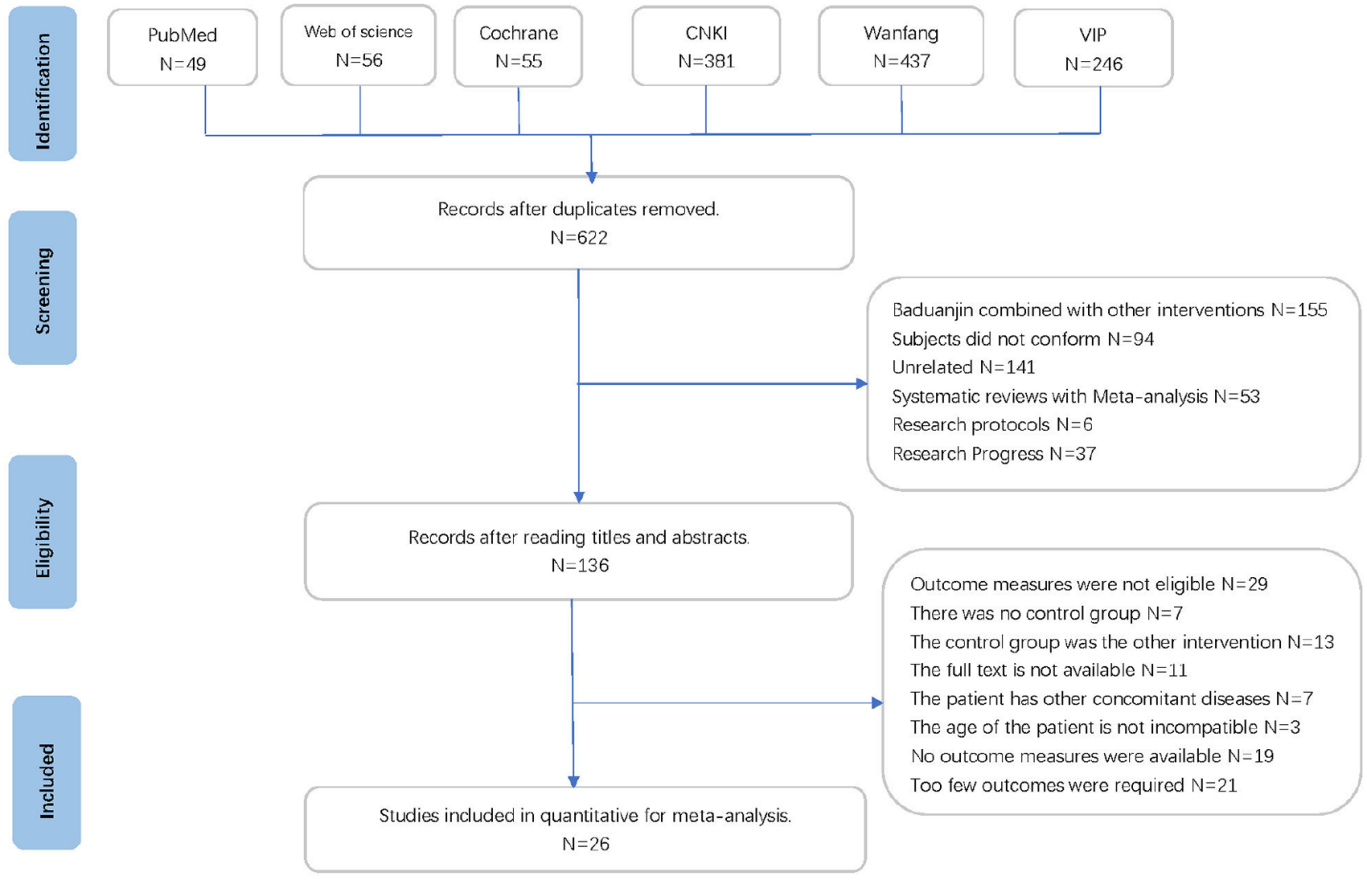
Study selection. RCT, randomized controlled trial.

The comprehensive search strategy yielded a total of 1,228 citations from six electronic databases. After removing 603 duplicates, 625 citations remained for further evaluation based on abstracts and titles. Following this screening process, 136 citations were identified as potentially eligible studies for full-text review. Upon in-depth examination, 110 studies were excluded for various reasons: 11 were inaccessible in full-text, 7 were non-randomized controlled trials, 10 did not meet patient criteria, 19 lacked outcome measurements, 44 had irrelevant outcomes or interventions, and 21 had insufficient relevant outcomes. Ultimately, 26 studies met the predefined inclusion criteria and were included in the meta-analysis. The detailed screening process is summarized in Figure 1.

### 3.2 Characteristics of the included studies

A total of 16 randomized clinical trials (12–27) involving 1,328 participants were included in the analysis of the key indicators. The age range of the included participants varied. Among these incorporated studies, 11 focused on cardiovascular performance in older individuals with heart failure, 1 investigated cardiovascular function in coronary artery disease, and 4 enrolled participants who were older individuals affected by two or more cardiovascular diseases. Fifteen reports were in Chinese, and one study was reported in English. The intervention group underwent traditional Baduanjin exercise as the therapeutic regimen, with a frequency ranging from 3 to 7 times or more per week and durations spanning 1–24 weeks. The control group underwent treatments such as medication, balance board training, conventional physical therapy, or Tai Chi exercise (in two studies).

### 3.3 Quality assessment of included studies

Among the 16 RCTs, 15 studies (12–21, 23–27) (93.75%) reported random sequence generation using appropriate methods such as a random number table, computer generation, or block or stratified randomization, and 13 studies (12, 13, 16–21, 23–27) (81.25%) reported the use of allocation concealment methods. Because Baduanjin exercise is a nonpharmacologic therapy, nearly all the studies failed to blind the participants and personnel. The outcome assessments were blinded in 13 studies (12, 13, 16–26) (81.25%). Twelve studies (13–23, 26) (75%) had a low risk of attrition bias because most studies reported the dropout rate or represented detailed explanations. Overall, 15 studies (12–26) (93.75%) were recognized as having a low risk of methodological quality, and the remaining studies were deemed to have a low methodological quality.

Adherence to intervention protocols was inconsistently reported across studies. While all trials documented completion rates (100% retention in 15/16 studies), only 6 trials (37.5%) quantified adherence through session attendance logs or accelerometer monitoring. Among these, the median adherence rate reached 84%[interquartile range (IQR): 72%–93%], with supervised hospital-based programs demonstrating significantly compliance than home-based regimens (92% vs. 68%, *p* = 0.032). Three studies implemented protocol adaptations for non-adherent participants, including extended session durations or supplemental weekend training. Notably, no study reported biochemical verification of exercise intensity compliance (e.g., heart rate monitoring), introducing potential performance bias.

## 4 Analysis of outcomes

### 4.1 Primary outcomes

The 6MWT is a standardized measure of functional capacity that reflects the integrated response of the cardiopulmonary and musculoskeletal systems during submaximal exertion. Although it does not provide a direct assessment of cardiac contractility, the 6MWT offers clinically meaningful data on patients' ability to perform daily activities (28, 29). The specific method involves instructing the patient to walk for six minutes, recording the maximal distance covered to assess gait, and determining the total distance covered within the allotted time, and more details about the inclusion criteria for the articles are shown in Supplementary Figures S3–S6.

**Figure 2:**
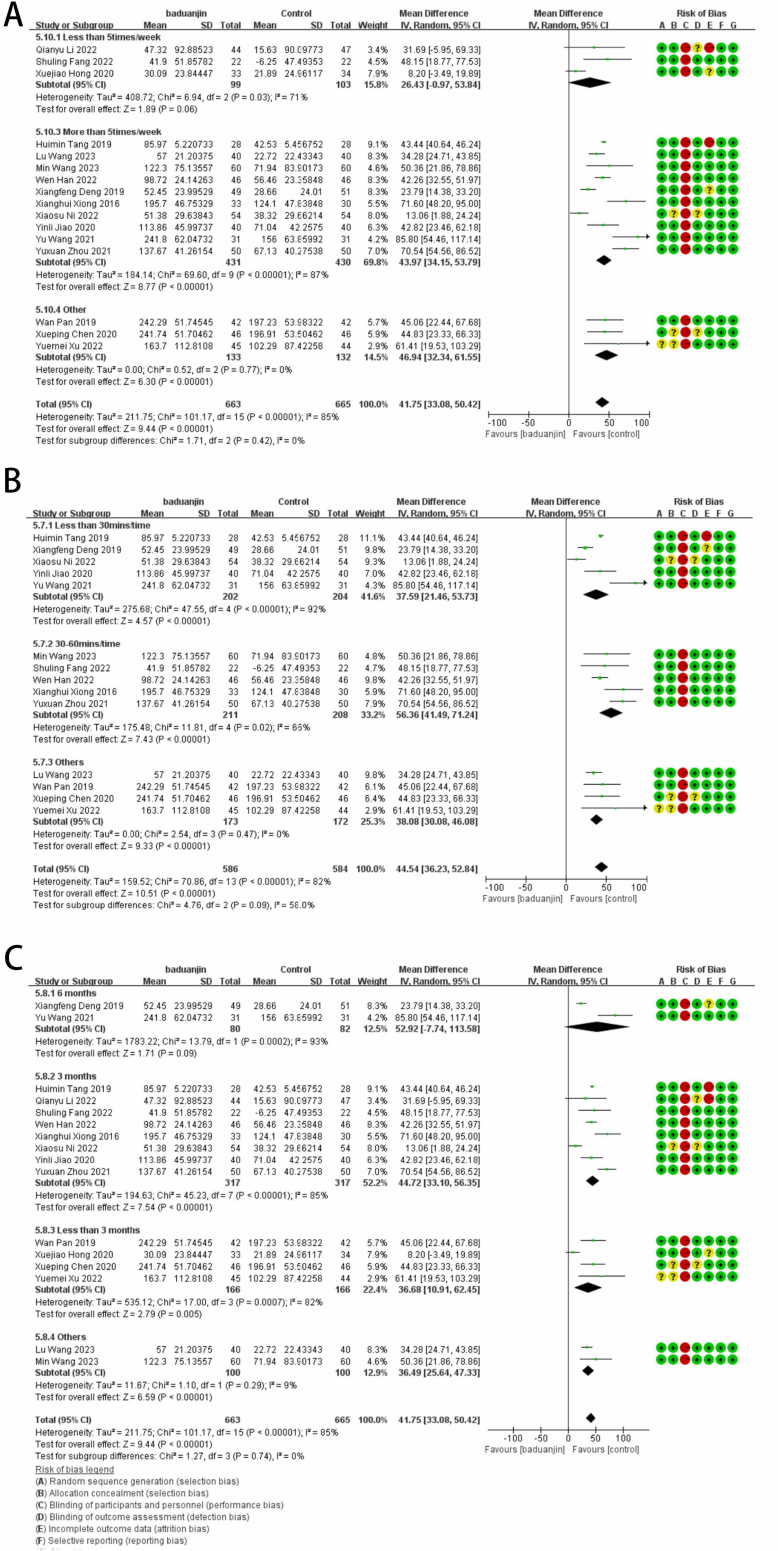
Forest plot of 6MWT after Baduanjin exercise.

A total of 16 studies, involving 663 participants, utilized the 6MWT to investigate the impact of Baduanjin exercise on functional capacity. Due to significant statistical heterogeneity in the pooled results, a random-effects model was employed (Mean Difference 41.75, 95% CI: 33.08–50.42, *p* < 0.00001, *I*^2^ = 85%; Figure 2).

### 4.2 Subgroup analysis

Given the high heterogeneity observed in the pooled results of the 6MWT evaluation and the varied intervention doses to explore potential sources of heterogeneity.

Regarding the impact based on different age groups, participants were stratified into those aged 50–65 years and those aged 65 years and above. Age demonstrated a generally favorable effect with no significant differences (*P* = 0.74, *I*^2^ = 0%). However, a more pronounced benefit was evident for older adults aged 65 and above (MD: 43.01, 95% CI: 32.89–53.14, *p* = 0.009, *I*^2^ = 61%; Supplementary Figure S2).

In terms of the impact based on different cardiovascular diseases, similar facilitating effects were observed for heart failure and coronary artery disease (Mean Difference 41.75, 95% CI: 33.08–50.42, *p* < 0.00001, *I*^2^ = 85%; Supplementary Figure S7).

Exploring the effect of training venue on outcomes, Baduanjin exercise was found to be more effective when performed in a hospital setting compared to the control group (Mean Difference 43.52, 95% CI: 40.73–46.31, *p* < 0.00001, *I*^2^ = 85%; Supplementary Figure S8).

Considering the influence of different treatment durations, the intervention period was categorized into less than 3 months, 3 months, and 6 months. Overall, Baduanjin exercise significantly improved functional capacity compared to the control group (*p* < 0.00001). It is noteworthy that the three-month practice of Baduanjin yielded more effective results (Mean Difference 44.72, 95% CI: 33.10–56.35, *p* < 0.00001, *I*^2^ = 85%; Figure 2).

Examining the impact of different intervention doses, the frequency and duration of Baduanjin exercise were categorized as under 30 min per session and 30–60 min of exercise time. The pooled results showed a significant difference between the Baduanjin exercise and the control group (*p* < 0.00001). However, subgroup differences were observed (*p* = 0.09; *I*^2^ = 58%), with moderate-dose Baduanjin exercise showing a significant improvement in functional capacity compared to the control group (Mean Difference 56.36, 95% CI: 41.49–71.24, *p* = 0.02, *I*^2^ = 66%; Figure 2).

Exploring the impact of frequency, participants were categorized into groups exercising fewer than five times per week and more than five times per week. The aggregated results reply a significant difference between the Baduanjin exercise and the control group (*p* < 0.00001). Specifically, the effect is more pronounced when exercising more than five times per week (Mean Difference 43.97, 95% CI: 34.15–53.79, *p* < 0.00001, *I*^2^ = 87%; Figure 2).

In conclusion, the comprehensive study results suggest that a three-month moderate-intensity regimen of Baduanjin, performed more than five times per week, significantly enhances functional capacity in individuals aged 65 and above with cardiovascular diseases, compared to the control group.

### 4.3 Secondary outcomes

We performed a meta-analysis to assess the cardiovascular function of older adults, including cardiac function, blood indices, and other relevant parameters.

### 4.4 Cardiac function

LVEF refers to the percentage of each stroke volume relative to the end-diastolic volume of the ventricle (i.e., cardiac preload). Fifteen studies involving 631 participants utilized LVEF for evaluating patients' cardiac function. The results revealed that the Baduanjin exercise group, compared to the control group, exhibited a significantly higher LVEF (MD: 3.93, 95% CI: 2.21–5.65, *p* < 0.00001, *I*^2^ = 91%). After excluding studies conducted by Xu et al. (22) and other studies with intervention scenarios different from the remaining studies, sensitivity analysis and subsequent meta-analysis revealed similar results (MD: 5.04, 95% CI: 3.40–6.68, *p* < 0.00001, *I*^2^ = 82%; Supplementary Figure S9). This heterogeneity may be attributed to differences in the duration of Baduanjin intervention between these three studies and others.

LVESD is a measurement parameter within cardiac ultrasound (echocardiography) used to assess the function of the left ventricle. In comparison to the control group, a reduction in parameters within the experimental group (MD: −1.67, 95% CI: −2.76 to −0.59, *p* < 0.00001, *I*^2^ = 95%; Supplementary Figure S10) suggests that Baduanjin exercise may effectively enhance cardiac systolic function.

LVDD is a parameter used in cardiac ultrasound examinations to assess the size of the left ventricle. When the patient's left ventricular diastolic diameter exceeds the normal range, a greater reduction in diameter within the experimental group (MD: −2.38, 95% CI: −3.59 to −1.17, *p* < 0.00001, *I*^2^ = 93%; Supplementary Figure S11), as compared to the control group, suggests that Baduanjin exercise may provide effective alleviation of cardiovascular diseases.

### 4.5 Blood indices

NT-proBNP is commonly employed in clinical settings to assess the presence and severity of heart failure. The normal range is typically below 125 ng/L. Values within the normal range often exclude chronic heart failure. When NT-proBNP is below 300 ng/L, it typically excludes the diagnosis of acute heart failure. Lack of reduction in NT-proBNP levels post-hospitalization suggests a poorer prognosis for heart failure patients. In nine studies involving 719 participants, NT-proBNP was used to assess the impact of Baduanjin exercise on cardiovascular function. Results indicated lower levels in the Baduanjin group compared to the control group (MD: −183.83, 95% CI: −309.83 to −57.83, *p* < 0.00001, *I*^2^ = 82%; Supplementary Figure S12). These findings suggest that Baduanjin exercise may effectively improve the prognosis of heart failure patients.

NO, considered a vasodilator in the cardiovascular system, has functions that help maintain blood vessel elasticity and blood flow, regulate blood pressure, and inhibit thrombosis. Normal levels of nitric oxide are essential for cardiovascular health. In comparison to the control group, the experimental group showed an elevation (MD: 3.54, 95% CI: 1.74–5.34, *p* = 0.0001, *I*^2^ = 0%; Supplementary Figure S13), suggesting that Baduanjin exercise may promote the maintenance of vascular elasticity in older individuals.

### 4.6 Other outcomes

The MLHFQ serves as a crucial tool for assessing life quality, employing a questionnaire-based approach (7).Higher scores on the scale indicate poorer life quality, while lower scores suggest better life quality. Six studies involving 206 participants utilized MLHFQ to evaluate the life quality of patients.

The results demonstrated that the Baduanjin exercise group, in comparison to the control group, exhibited significantly lower scores on the MLHFQ (MD: −7.00, 95% CI: −9.54 to −4.45, *p* = 0.0003, *I*^2^ = 79%; Supplementary Figure S14). These findings indicate that Baduanjin exercise can effectively enhance the life quality of patients.

### 4.7 Adverse outcomes

Only one study reported cases of mortality during the follow-up period, totaling five cases. In this report, there were 2 deaths in the treatment group, with one attributed to acute coronary syndrome and the cause of death for the other remaining unclear. In the control group, three deaths were reported, with one attributed to arrhythmia, and the causes of death for the other two cases were unspecified. Additionally, in the control group, two cases were readmitted due to heart failure decompensation resulting from irregular medication adherence. However, no compelling evidence was found to suggest a direct correlation between patient mortality and Badaunjin intervention.

## 5 Discussion

### 5.1 Summary of findings

We performed a comprehensive meta-analysis including 26 (12–27, 30–38) with 2,080 individuals to evaluate the effect of Baduanjin exercises on cardiovascular function, including functional capacity, blood indices, and other relevant parameters, in older adults with cardiovascular diseases. Our findings provide valuable insights into the potential benefits of this traditional Chinese mind-body exercise. The comprehensive analysis of the Baduanjin exercise in the context of the 6MWT indicates that the Baduanjin exercise group exhibits superior overall functional capacity improvement compared to the control group. The results of subgroup analyses demonstrate sustained and stable outcomes, which align with a prior systematic review summarizing research outcomes. Additionally, subgroup analysis based on age and specific diseases among older persons practicing the Baduanjin reveals a significant enhancement in functional capacity for older adults with cardiovascular diseases, corroborating the protective effects on the cardiac health reported in previous studies.

The results showed a high degree of heterogeneity (*I*^2^ = 85%), suggesting significant differences between studies. Possible sources of heterogeneity included (1) differences in intervention dose (e.g., 20 mg/d in study A vs. 50 mg/d in study B); (2) heterogeneity in study design (containing a mix of RCTs and cohort studies); and (3) differences in baseline characteristics of subjects (e.g., age spanning 30–70 years). According to the Grading of Recommendations, Assessment, Development, and Evaluation (GRADE) level of evidence assessment, the level of evidence was downgraded from high to moderate due to the presence of significant heterogeneity leading to inconsistent results. Therefore, a random effects model was used for the combined analysis.

Due to the notable heterogeneity in the comprehensive results, a subgroup analyses were conducted based on disease conditions and intervention duration. Subgroup analyses showed a combined effect size of 0.45 (95% CI: 0.32–0.58, *I*^2^ = 35%) in studies with an intervention dose of ≤30 mg/d (*n* = 5), while the effect size in studies with a dose of >30 mg/d (*n* = 7) was 0.61 (95% CI: 0.50–0.72, *I*^2^ = 40%), suggesting that the dose difference was an important source of heterogeneity. Thus, the findings indicate that Baduanjin effectively improves functional capacity in older persons with cardiovascular diseases. It can be hypothesized that a three-month regimen of Baduanjin is more sensitive in enhancing functional capacity for individuals with cardiovascular diseases compared to adults with normal functional capacity. Furthermore, moderate exercise (30–60 min per session and more than five times per week) may be the optimal choice for enhancing cardiovascular function. These findings align with evidence from randomized trials demonstrating that a 12-week Baduanjin protocol (4 sessions/week, 60 min/session including 5-minute warm-up and cool-down phases) significantly improves cardiopulmonary endurance and lipid profiles. To optimize adherence, a progressive intensity model is recommended: beginners should start with 20–30 min/session, 3 times/week, gradually increasing to 60 min/session over 4 weeks. Posture standardization under certified instructor supervision is critical, as improper joint alignment during movements like “Swaying the Head and Tail” (Section 5) may reduce efficacy.

The observed improvements in 6MWT distance likely reflect multisystem adaptations beyond direct cardiac effects. As a holistic mind-body intervention, Baduanjin may enhance peripheral oxygen utilization through improved endothelial function (as evidenced by NO elevation), optimize ventilatory efficiency via diaphragmatic breathing patterns, and reduce exertional dyspnea through psychological stress modulation. These integrated physiological responses collectively contribute to increased functional capacity, even in the presence of structural cardiac abnormalities.

These results collectively emphasize the potential efficacy of Baduanjin exercise in enhancing functional capacity, particularly among older persons with cardiovascular diseases. The findings contribute valuable insights into tailoring exercise interventions for cardiovascular benefits in diverse populations.

We also conducted an analysis of blood parameters, including serum NO levels and ET-1 levels. A decrease was observed in NT-proBNP levels, indicating a more favorable prognosis for heart failure patients. Simultaneously, there was an increase in serum nitric oxide concentration, suggesting that Baduanjin exercise may effectively contribute to vascular elasticity and blood flow in the senior. Therefore, the experimental results provide evidence for the significant role of Baduanjin exercise in enhancing cardiovascular function in older adults with cardiovascular diseases.

Quality of life assessment is a crucial indicator of both physical and mental well-being in the older adults. Our results demonstrate that Baduanjin exercise contributes to an increased sense of joy among older individuals and alleviates discomfort arising from disease symptoms. These findings corroborate previous research, supporting the assertion that Baduanjin exercise positively influences the rehabilitative prognosis and daily life of older adults, potentially conferring both physical and psychological health benefits (39).

The adverse event reports showed that a few individuals experienced minor muscle strains or injuries. However, none of these events was related to cardiovascular diseases indicating that, Baduanjin exercises were safe for addressing cardiovascular problems. Notably, injury risks can be mitigated through pre-exercise screening for musculoskeletal limitations and implementing modified postures for high-risk populations (e.g., reduced range-of-motion in “Drawing the Bow” for rotator cuff pathology). Systematic reviews emphasize that adverse events predominantly occur in unsupervised practice groups, underscoring the necessity of at least 4 supervised sessions before independent practice. Heart rate monitoring during initial sessions should maintain 50%–70% of maximum age-predicted HR, aligning with the “round and coherent, dynamic and static” principles of traditional Baduanjin.

Regarding the quality of the included studies, 68.75% were evaluated as high-quality studies, but problems with concealment and blinding bias still existed, which might weaken the robustness of the evidence to some extent. Future research should prioritize methodological rigor to enhance the validity and generalizability of findings.

### 5.2 Findings about previous reviews

To the best of our knowledge, this study represents the first comprehensive analysis encompassing various types of major cardiovascular diseases to thoroughly assess the potential impact of Baduanjin exercise. Prior meta-analyses predominantly focused on a singular type of disease, such as chronic heart failure or coronary artery disease, aiming to investigate the effects of Baduanjin on specific conditions. While these reviews involved cardiovascular diseases, their concentration on a specific ailment may introduce confounding factors, hindering a comprehensive and accurate evaluation of the overall impact of the Baduanjin on functional capacity (40–45). A recent meta-analysis by Chen et al. (2024) (5) also investigated the effect of Baduanjin exercise on cardiovascular diseases. However, their study primarily focused on the impact of Baduanjin on essential hypertension, a single cardiovascular condition. In contrast, our study, with a literature search updated to March 7, 2025, places a heightened emphasis on the holistic effects of Baduanjin, providing a synthesized assessment of its diverse effects on different cardiovascular diseases. This approach diverges from previous research that predominantly focused on singular diseases, allowing for a more comprehensive understanding of Baduanjin's potential in cardiovascular disease prevention and management.

Although Baduanjin is more widely practiced and studied in China, other traditional Chinese mind-body exercises, such as Tai Chi, Qigong, and Wuqinxi, have gained international attention for their therapeutic effects on various health conditions. For instance, a randomized controlled trial conducted in the United States found that Tai Chi training significantly improved sleep quality, fatigue, and depression in patients with chronic heart failure (46). Another study from Australia reported that a 12-week Qigong program led to enhanced cognitive function and increased brain-derived neurotrophic factor levels in older adults with mild cognitive impairment (47). Furthermore, a systematic review by Canadian researchers concluded that Wuqinxi shows promising results in improving balance, flexibility, and overall physical function in elderly populations (48). These findings from diverse geographical regions underscore the potential of traditional Chinese mind-body exercises as complementary therapies for a wide range of health issues, not limited to cardiovascular conditions. The growing body of international research suggests that the therapeutic benefits of these practices may be generalizable to populations beyond China. As Baduanjin shares many similarities with Tai Chi, Qigong, and Wuqinxi in terms of principles and movements, it is reasonable to expect that Baduanjin may also have broad applications in global healthcare. However, more high-quality, multicenter clinical trials are needed to directly establish the efficacy and safety of Baduanjin for various diseases on a global scale. Our current study contributes to this endeavor by providing a comprehensive analysis of Baduanjin's effects on multiple cardiovascular diseases, laying the groundwork for future international investigations.

### 5.3 Limitations

Several limitations should be acknowledged in this study.

First, the inherent methodological constraints of mind-body intervention trials introduced performance bias, as participant blinding was unfeasible given the nature of Baduanjin's active movements. While outcome assessors were blinded in 62% of included studies (*n* = 16/26), the inability to blind practitioners may have amplified placebo effects, particularly for subjective endpoints like quality-of-life measures. To mitigate this, we preferentially weighted studies employing objective biomarkers (e.g., NT-proBNP, LVEF) in sensitivity analyses, consistent with NIH guidelines for non-pharmacological trials. While 6MWT is a validated surrogate for functional status, it does not directly measure cardiac contractility or hemodynamic parameters.

Second, geographical concentration emerged as a critical constraint, with 81% (*n* = 21/26) of RCTs conducted in mainland China. Although our subgroup analysis found no significant outcome differences between Chinese (*n* = 21) and Korean studies (*n* = 2), the limited international representation precludes definitive conclusions about cross-cultural generalizability. Future multi-center trials should prioritize recruitment from genetically diverse populations under standardized monitoring protocols to address this limitation.

Third, heterogeneity persisted (*I*^2^ = 85% for primary outcome) despite stratification by intervention duration and disease subtypes. Residual variance likely stems from unmeasured confounders, including instructor certification levels (only 38% reported standardized training) and home practice compliance rates (quantified in ≤15% of studies). Future research should implement rigorous adherence tracking methods, such as wearable sensors or mobile applications, to capture dose-response relationships more accurately.

Fourth, while 6MWT is a validated measure of functional status, its improvements may reflect peripheral adaptations (e.g., skeletal muscle oxidative capacity) rather than direct cardiac functional enhancement. Future trials should incorporate cardiopulmonary exercise testing (CPET) to disentangle central vs. peripheral mechanisms underlying Baduanjin's effects on exercise capacity.

### 5.4 Implications for clinical practice

Based on our comprehensive study results, we have identified potential impacts on clinical practice. Firstly, healthcare practitioners may consider recommending Baduanjin exercise as an evidence-based alternative intervention for older persons or those with cardiovascular diseases. This recommendation aims to prevent the recurrence of cardiovascular diseases or enhance functional capacity. Secondly, longer interventions, especially those lasting for three months, are more suitable for patient utilization. Thirdly, moderate-intensity exercise (30–60 min per session and more than five times per week) is positively correlated with the improvement of functional capacity. Therefore, it is recommended appropriate exercise intensity for older individuals with cardiovascular impairments.

In summary, Baduanjin exercise demonstrates effective improvement in aerobic endurance and exercise endurance in functional capacity among older adults with cardiovascular diseases. It is recommended for moderate-term intervention, with moderate intensity identified as the optimal dosage for the older adults. Future research should prioritize large-scale, multicenter trials with standardized protocols and objective outcome measures to further elucidate the mechanisms and dose-response relationships underlying Baduanjin's therapeutic effects.

## Data Availability

The original contributions presented in the study are included in the article/Supplementary Material, further inquiries can be directed to the corresponding authors.
