# Supplementary material for: Baduanjin exercise improves functional capacity and cardiovascular function in older adults with cardiovascular diseases: a systematic review and meta-analysis
Source: Front Cardiovasc Med. 2025 Apr 24;12:1419095. doi: 10.3389/fcvm.2025.1419095 (PMC12058754; doi:10.3389/fcvm.2025.1419095)
Supplement: Supplementary file 1 [file Presentation1.pdf]

Supplementary Content

Supplementary Methods S1 Search strategy..1

Supplementary Methods S2 Formulae for combining groups...2

Supplementary Figure S1Risk of bias summary..3

Supplementary Figure S2 Forest plot of comparison: Age..6

Supplementary Figure S3 Forest plot of comparison: female..7

Supplementary Figure S4Forest plot of comparison: male..8

Supplementary Figure S5 Risk of bias graph...9

Supplementary Figure S6 Funnel plot of comparison: Cardiac function, outcome: minute walk test...10

Supplementary Figure S7Forest plot of comparison: Subgroups analysis, outcome: Disease 11

Supplementary Figure S8Forest plot of comparison: Subgroups analysis, outcome: Exercise Facility..12

Supplementary Figure S9Forest plot of comparison: Cardiac function, outcome: LVEF …..13

Supplementary Figure S10Forest plot of comparison: Cardiac function, outcome: LVESD...14

Supplementary Figure S11Forest plot of comparison: Cardiac function, outcome: LVDD..15

Supplementary Figure S12 Forest plot of comparison: Cardiac function, outcome: NT-proBNP...16

Supplementary Figure S13Forest plot of comparison: Cardiac function, outcome: serum NO...17

Supplementary Figure S14 Forest plot of comparison: Others, outcome: MLHFQ...18

Supplementary Table S1 Characteristics of included studies..19

Supplementary Table S2 PRISMA 2020 Checklist..25

Supplementary Table S3 Grade evidence profile of cardiovascular outcomes and adverse events...28

1.supplementary Methods S1:Searchstrategy

PubMed search strategy

#1 Baduanjin[Title/Abstract]

#2 Ba duanjin[Title/Abstract]

#3 Ba duan jin[Title/Abstract]

#4 Eight-section Brocade[Title/Abstract]

#5 Eight trigrams boxing[Title/Abstract]

#6 #1 OR #2 OR #3 OR #4 OR #5

#7 " **Cardiac function** "[Mesh]

#8 **heart function** [Title/Abstract]

#9 cardiac failure [Title/Abstract]

#10 **heart failure** [Title/Abstract]

#11 **heart attacks** [Title/Abstract]

#12 hypertension Title/Abstract]

#13 highblood pressure [Title/Abstract]

#14 ferritin [Title/Abstract]

#15 coronary artery heart disease [Title/Abstract]

#16 **myocardial infarction** [Title/Abstract]

#17 stenocardia [Title/Abstract]

#18 **angor pectoris**[Title/Abstract]

#19 CHF [Title/Abstract]

#20 CHD [Title/Abstract]

#21 CAD[Title/Abstract]

#22 HTN [Title/Abstract]

#23 MI [Title/Abstract]

#24 AP [Title/Abstract]

#25 HF[Title/Abstract]

#26 #7 OR #8 OR #9 OR #10 OR #11 OR #12 OR #13 OR #14 OR #15 OR #16 OR #17 OR #18 OR #19 OR #20 OR #21 OR #22 OR #23 OR #24 OR #25

#27 random*[Title/Abstract]

#28 older adults [Title/Abstract]

#29 #6 AND #26 AND #27 AND #28

("**Cardiac function** "[MeSH Terms] OR " **heart function** "[Title/Abstract] OR " cardiac failure "[Title/Abstract] OR " **heart failure** "[Title/Abstract] OR " **heart attacks** "[Title/Abstract] OR " hypertension "[Title/Abstract] OR " highblood pressure "[Title/Abstract] OR " ferritin "[Title/Abstract] OR " coronary artery heart disease "[Title/Abstract] OR " **myocardial infarction**"[Title/Abstract] OR " stenocardia "[Title/Abstract] OR"**angor pectoris** " [Title/Abstract] OR "CHF " [Title/Abstract] OR " CHD " [Title/Abstract] OR " CAD " [Title/Abstract] OR " HTN " [Title/Abstract] OR" MI " [Title/Abstract] OR" AP " [Title/Abstract] OR "HF " [Title/Abstract]) AND ("Ba duanjin"[Title/Abstract] OR "Baduanjin"[Title/Abstract] OR "ba duan jin"[Title/Abstract] OR "eight section brocade"[Title/Abstract] OR "Eight trigrams boxing" [Title/Abstract]) AND （ "random*"[Title/Abstract]） AND "older adults" [Title/Abstract]

Web of science **search strategy**
#1 Baduanjin[Topic]
#2 Ba duanjin[Topic]
#3 Ba duan jin[Topic]
#4 Eight-section Brocade[Topic]
#5 Eight trigrams boxing[Topic]
#6 #1 OR #2 OR #3 OR #4 OR #5
#7 " **Cardiac function** "[Topic]
#8 **heart function**[Topic]
#9 cardiac failure [Topic]
#10 **heart failure** [Topic]
#11 **heart attacks** [Topic]
#12 hypertension [Topic]
#13 highblood pressure[Topic]
#14 ferritin [Topic]
#15 coronary artery heart disease[Topic]
#16 **myocardial infarction**[Topic]
#17 stenocardia[Topic]]
#18 **angor pectoris**[Topic]
#19 CHF [Topic]
#20 CHD [Topic]
#21 CAD[Topic]
#22 HTN [Topic]
#23 MI [Topic]
#24 AP[Topic]
#25 HF[Topic]
#26 #7 OR #8 OR #9 OR #10 OR #11 OR #12 OR #13 OR #14 OR #15 OR #16 OR #17 OR #18 OR #19 OR #20 OR #21 OR #22 OR #23 OR #24 OR #25
#27 random*[Topic]
#28 older adults[Topic]
#29 #6 AND #26 AND #27 AND #28
((TS= （ random*)) AND((TS=older adults)) AND (((((((((((((((TS=(**Cardiac function**) OR TS=**(heart function**))OR TS=(cardiac failure))OR TS=(**heart failure**))OR TS=(**heart attacks**)) OR TS=(hypertension))OR TS=(highblood pressure)) OR TS=(ferritin)) OR TS=(coronary artery heart disease)) OR TS=(**myocardial infarction**)) OR TS=(stenocardia)) OR TS=**angor pectoris**)) OR TS=(CHF)) OR TS=(CHD)) OR TS=(CAD)) OR TS=(HTN)) OR TS= MI))OR TS=(AP)) OR TS=(HF))AND ((((((TS=Ba duanjin)) OR TS=Baduanjin)) OR TS=(ba duan jin)) OR TS=(eight section brocade)) OR TS=Eight trigrams boxing))

Cochrane Library **search strategy**
#1 Baduanjin[[Title Abstract Keyword]
#2 Ba duanjin[Title Abstract Keyword]
#3 Ba duan jin[Title Abstract Keyword]
#4 Eight-section Brocade[Title Abstract Keyword]

#5 Eight trigrams boxing[Title Abstract Keyword]
#6 #1 OR #2 OR #3 OR #4 OR #5
#7 **Cardiac function** [Title Abstract Keyword]
#8 **heart function**[Title Abstract Keyword]
#9 cardiac failure [Title Abstract Keyword]
#10 **heart failure** [Title Abstract Keyword]
#11 **heart attacks** [Title Abstract Keyword]
#12 hypertension [Title Abstract Keyword]
#13 highblood pressure[Title Abstract Keyword]
#14 ferritin [Title Abstract Keyword]
#15 coronary artery heart disease[Title Abstract Keyword]
#16 **myocardial infarction**[Title Abstract Keyword]
#17 stenocardia[Title Abstract Keyword]
#18 **angor pectoris**[Title Abstract Keyword]
#19 CHF [Title Abstract Keyword]
#20 CAD[Title Abstract Keyword]
#21 HTN [Title Abstract Keyword]
#22 MI [Title Abstract Keyword]
#23 AP[Title Abstract Keyword]
#24 HF[Title Abstract Keyword]
#25 CHD[Title Abstract Keyword]
#26 #7 OR #8 OR #9 OR #10 OR #11 OR #12 OR #13 OR #14 OR #15 OR #16 OR #17 OR #18 OR #19 OR #20 OR #21 OR #22 OR #23 OR #24 OR #25
#27 random*[Title Abstract Keyword]
#28 older adults[Title Abstract Keyword]
#29 #6 AND #26 AND #27 AND #28

2. supplementary Methods S2: Formulae for combining groups

The means and SD were combined using formulae for continuous outcomes referred to Chapter 6: Choosing effect measures and computing estimates of effect. In: Higgins JPT, Thomas J, Chandler J, Cumpston M, Li T, Page MJ, Welch VA (editors). Cochrane Handbook for Systematic Reviews of Interventions version 6.2 (updated February 2021)

Formulae for combining summary statistics across two groups: Group 1 (with sample size = N1, mean = M1 and SD = SD1) and Group 2 (with sample size = N2, mean = M2 and SD = SD2)

	Combined groups
Sample size	$N1 + N2$
Mean	$\frac{N_1M_1 + N_2M_2}{N_1 + N_2}$
SD	$\sqrt{\frac{(N_1 - 1)SD_1^2 + (N_2 - 1)SD_2^2 + \frac{N_1N_2}{N_1 + N_2}(M_1^2 + M_2^2 - 2M_1M_2)}{N_1 + N_2 - 1}}$

When there are more than two groups to combine, the simplest strategy is to apply the above formula sequentially (i.e. combine Group 1 and Group 2 to create Group '1+2', then combine Group '1+2' and Group 3 to create Group '1+2+3', and so on).

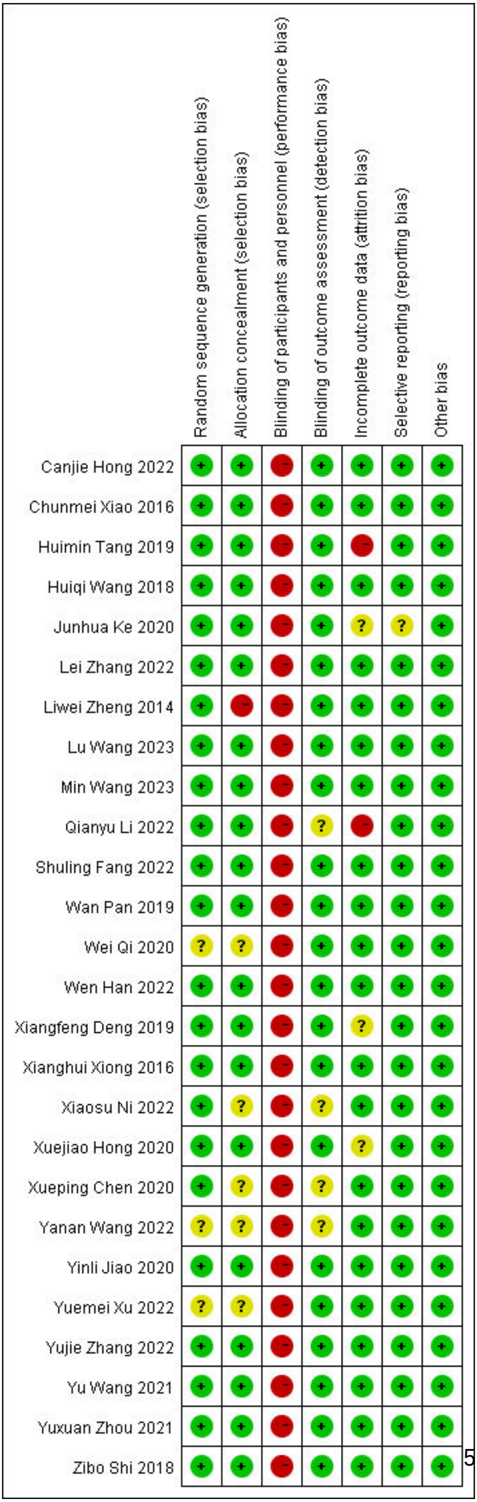

Risk of bias summary: review authors' judgements about each risk of bias item for each included study.

Study or Subgroup	baduanjin Mean	baduanjin SD	baduanjin Total	Control Mean	Control SD	Control Total	Weight	Mean Difference IV, Random, 95% CI
**5.2.1 Aged from 50 to 65**								
Huimin Tang 2019	85.97	5.220733	28	42.53	5.456752	28	9.1%	43.44 [40.64, 46.24]
Qianyu Li 2022	47.32	92.88523	44	15.63	90.09773	47	3.4%	31.69 [-5.95, 69.33]
Wen Han 2022	98.72	24.14263	46	56.46	23.35848	46	8.3%	42.26 [32.55, 51.97]
Xiaosu Ni 2022	51.38	29.63843	54	38.32	29.66214	54	8.0%	13.06 [1.88, 24.24]
Xuejiao Hong 2020	30.09	23.84447	33	21.89	24.96117	34	7.9%	8.20 [-3.49, 19.89]
Yu Wang 2021	241.8	62.04732	31	156	63.85992	31	4.2%	85.80 [54.46, 117.14]
Yuxuan Zhou 2021	137.67	41.26154	50	67.13	40.27538	50	7.0%	70.54 [54.56, 86.52]
**Subtotal (95% CI)**			**286**			**290**	**47.9%**	**39.98 [24.73, 55.22]**
Heterogeneity: Tau² = 340.73; Chi² = 78.42, df = 6 (P < 0.00001); I² = 92%								
Test for overall effect: Z = 5.14 (P < 0.00001)								
**5.2.2 Aged above 65**								
Lu Wang 2023	57	21.20375	40	22.72	22.43343	40	8.3%	34.28 [24.71, 43.85]
Min Wang 2023	122.3	75.13557	60	71.94	83.90173	60	4.6%	50.36 [21.86, 78.86]
Shuling Fang 2022	41.9	51.85782	22	-6.25	47.49353	22	4.5%	48.15 [18.77, 77.53]
Wan Pan 2019	242.29	51.74545	42	197.23	53.98322	42	5.7%	45.06 [22.44, 67.68]
Xiangfeng Deng 2019	52.45	23.99529	49	28.66	24.01	51	8.3%	23.79 [14.38, 33.20]
Xianghui Xiong 2016	195.7	46.75329	33	124.1	47.83848	30	5.5%	71.60 [48.20, 95.00]
Xueping Chen 2020	241.74	51.70462	46	196.91	53.50462	46	5.9%	44.83 [23.33, 66.33]
Yinli Jiao 2020	113.86	45.99737	40	71.04	42.2575	40	6.3%	42.82 [23.46, 62.18]
Yuemei Xu 2022	163.7	112.8108	45	102.29	87.42258	44	2.9%	61.41 [19.53, 103.29]
**Subtotal (95% CI)**			**377**			**375**	**52.1%**	**43.01 [32.89, 53.14]**
Heterogeneity: Tau² = 123.56; Chi² = 20.26, df = 8 (P = 0.009); I² = 61%								
Test for overall effect: Z = 8.33 (P < 0.00001)								
**Total (95% CI)**			**663**			**665**	**100.0%**	**41.75 [33.08, 50.42]**
Heterogeneity: Tau² = 211.75; Chi² = 101.17, df = 15 (P < 0.00001); I² = 85%								
Test for overall effect: Z = 9.44 (P < 0.00001)								
Test for subgroup differences: Chi² = 0.11, df = 1 (P = 0.74), I² = 0%								

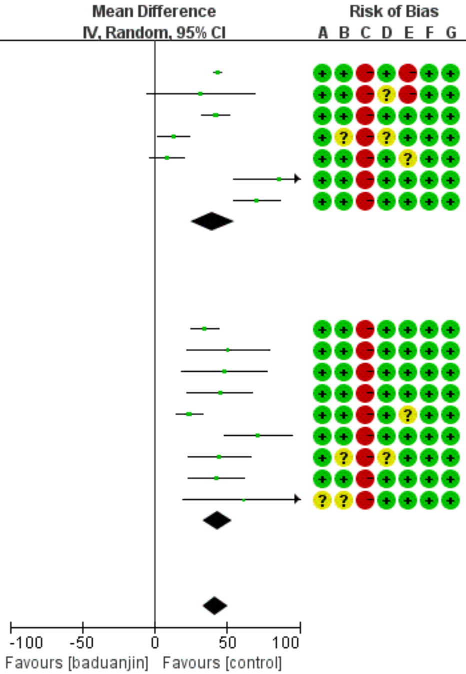

Risk of bias legend
(A) Random sequence generation (selection bias)
(B) Allocation concealment (selection bias)
(C) Blinding of participants and personnel (performance bias)
(D) Blinding of outcome assessment (detection bias)
(E) Incomplete outcome data (attrition bias)
(F) Selective reporting (reporting bias)
(G) Other bias

Forest plot of comparison: 5 Subgroups analysis, outcome: 5.2 Age.

1.3 female

Study or Subgroup	Baduanjin Events	Baduanjin Total	Control Events	Control Total	Weight	Odds Ratio M-H, Random, 95% CI	A	B	C	D	E	F	G
Canjie Hong 2022	30	60	29	60	6.6%	1.07 [0.52, 2.19]	+	+	-	+	+	+	+
Huimin Tang 2019	7	30	8	30	2.5%	0.84 [0.26, 2.70]	+	+	-	+	-	+	+
Junhua Ke 2020	9	30	14	30	3.0%	0.49 [0.17, 1.41]	+	+	-	+	?	?	+
Lei Zhang 2022	16	40	18	40	4.3%	0.81 [0.34, 1.98]	+	+	-	+	+	+	+
Liwei Zheng 2014	14	27	12	28	3.0%	1.44 [0.50, 4.16]	+	-	-	+	+	+	+
Lu Wang 2023	19	40	17	40	4.3%	1.22 [0.51, 2.96]	+	+	-	+	+	+	+
Min Wang 2023	27	60	26	60	6.5%	1.07 [0.52, 2.20]	+	+	-	+	+	+	+
Qianyu Li 2022	17	44	13	47	4.3%	1.65 [0.68, 3.98]	+	+	-	?	-	+	+
Shuling Fang 2022	13	22	9	22	2.3%	2.09 [0.63, 6.94]	+	+	-	+	+	+	+
Wan Pan 2019	20	42	19	42	4.6%	1.10 [0.47, 2.59]	+	+	-	+	+	+	+
Wei Qi 2020	16	50	13	50	4.5%	1.34 [0.56, 3.19]	?	?	-	+	+	+	+
Wen Han 2022	20	46	21	46	5.0%	0.92 [0.40, 2.08]	+	+	-	+	+	+	+
Xiangfeng Deng 2019	21	49	20	51	5.3%	1.16 [0.52, 2.58]	+	+	-	+	?	+	+
Xianghui Xiong 2016	13	33	12	30	3.3%	0.97 [0.35, 2.68]	+	+	-	+	+	+	+
Xiaosu Ni 2022	24	54	25	54	5.9%	0.93 [0.43, 1.98]	+	?	-	?	+	+	+
Xuejiao Hong 2020	10	33	9	34	3.0%	1.21 [0.42, 3.50]	+	+	-	+	?	+	+
Xueping Chen 2020	22	46	21	46	5.0%	1.09 [0.48, 2.48]	+	?	-	?	+	+	+
Yanan Wang 2022	24	45	22	45	4.9%	1.19 [0.52, 2.73]	?	?	-	?	+	+	+
Yinli Jiao 2020	22	40	21	40	4.4%	1.11 [0.46, 2.66]	+	+	-	+	+	+	+
Yuemei Xu 2022	21	45	19	44	4.8%	1.15 [0.50, 2.66]	?	?	-	+	+	+	+
Yujie Zhang 2022	15	40	18	40	4.2%	0.73 [0.30, 1.79]	+	+	-	+	+	+	+
Yuxuan Zhou 2021	23	50	19	50	5.3%	1.39 [0.63, 3.08]	+	+	-	+	+	+	+
Zibo Shi 2018	14	26	12	29	3.0%	1.65 [0.57, 4.81]	+	+	-	+	+	+	+
**Total (95% CI)**		**952**		**958**	**100.0%**	**1.10 [0.92, 1.32]**							
Total events	417		397										

Heterogeneity: $Tau^2 = 0.00$; $Chi^2 = 7.50$, df = 22 (P = 1.00); $I^2 = 0\%$

Test for overall effect: Z = 1.04 (P = 0.30)

7

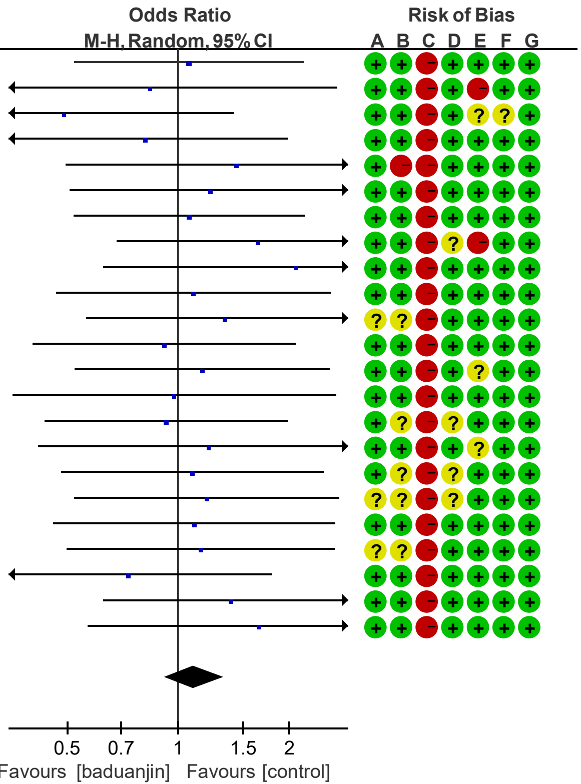

1.2 male

Study or Subgroup	Baduanjin Events	Baduanjin Total	Control Events	Control Total	Weight	Odds Ratio M-H, Random, 95% CI
Canjie Hong 2022	30	60	31	60	6.3%	0.94 [0.46, 1.91]
Huimin Tang 2019	23	30	22	30	2.4%	1.19 [0.37, 3.85]
Junhua Ke 2020	21	30	16	30	2.9%	2.04 [0.71, 5.89]
Lei Zhang 2022	24	40	22	40	4.1%	1.23 [0.51, 2.98]
Liwei Zheng 2014	13	27	16	28	2.9%	0.70 [0.24, 2.02]
Lu Wang 2023	21	40	23	40	4.2%	0.82 [0.34, 1.97]
Min Wang 2023	33	60	34	60	6.2%	0.93 [0.45, 1.92]
Qianyu Li 2022	27	44	34	47	4.2%	0.61 [0.25, 1.47]
Shuling Fang 2022	9	22	13	22	2.2%	0.48 [0.14, 1.59]
Wan Pan 2019	22	42	23	42	4.4%	0.91 [0.39, 2.14]
Wei Qi 2020	34	50	37	50	4.3%	0.75 [0.31, 1.78]
Wen Han 2022	26	46	25	46	4.8%	1.09 [0.48, 2.49]
Xiangfeng Deng 2019	28	49	31	51	5.1%	0.86 [0.39, 1.91]
Xianghui Xiong 2016	20	33	18	30	3.2%	1.03 [0.37, 2.82]
Xiaosu Ni 2022	30	54	29	54	5.6%	1.08 [0.51, 2.30]
Xuejiao Hong 2020	23	33	25	34	2.9%	0.83 [0.29, 2.40]
Xueping Chen 2020	24	46	25	46	4.8%	0.92 [0.40, 2.08]
Yanan Wang 2022	21	45	23	45	4.7%	0.84 [0.37, 1.91]
Yinli Jiao 2020	18	40	19	40	4.2%	0.90 [0.38, 2.18]
Yu Wang 2021	16	40	19	40	4.1%	0.74 [0.30, 1.79]
Yuemei Xu 2022	24	45	25	44	4.6%	0.87 [0.38, 2.00]
Yujie Zhang 2022	25	40	22	40	4.1%	1.36 [0.56, 3.33]
Yuxuan Zhou 2021	27	50	31	50	5.1%	0.72 [0.32, 1.60]
Zibo Shi 2018	12	26	17	29	2.8%	0.61 [0.21, 1.76]
**Total (95% CI)**		**992**		**998**	**100.0%**	**0.90 [0.75, 1.08]**
Total events	551		580			

Heterogeneity: Tau² = 0.00; Chi² = 7.71, df = 23 (P = 1.00); I² = 0%

Test for overall effect: Z = 1.16 (P = 0.25)

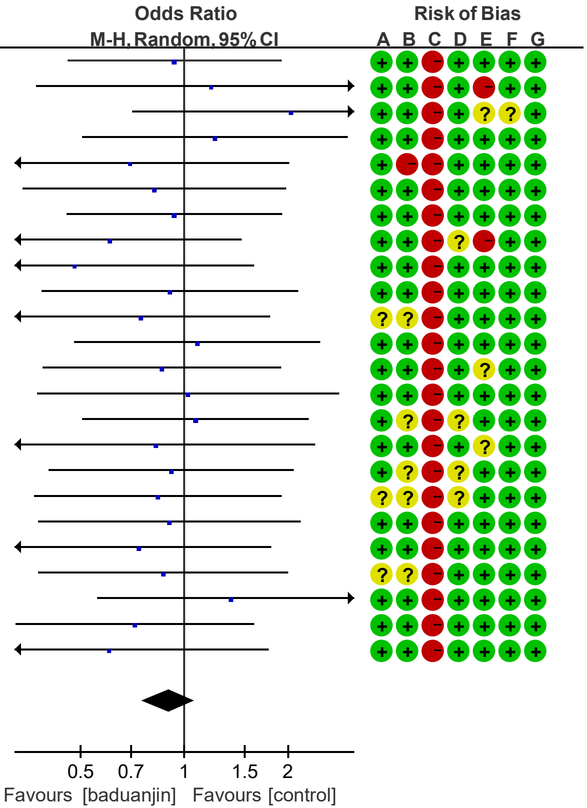

Figure 1

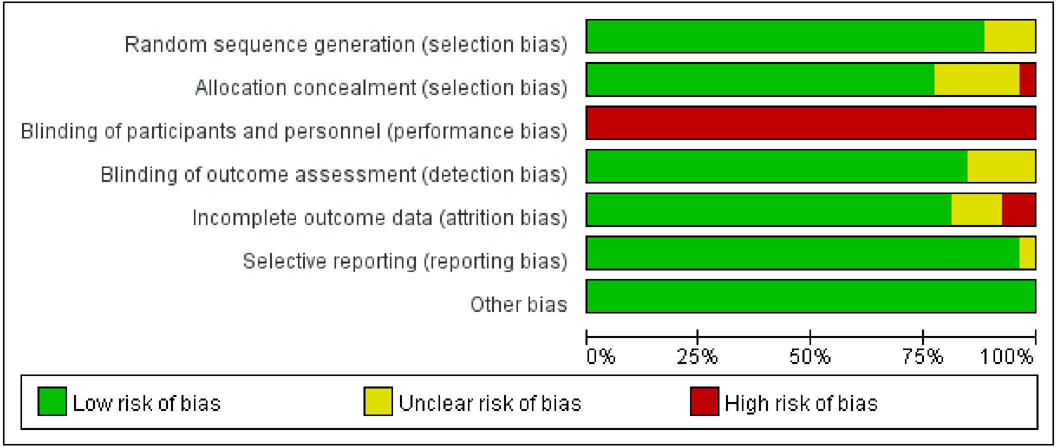

Risk of bias graph: review authors' judgements about each risk of bias item presented as percentages across all included studies.

Figure 10 (Analysis 2.3)

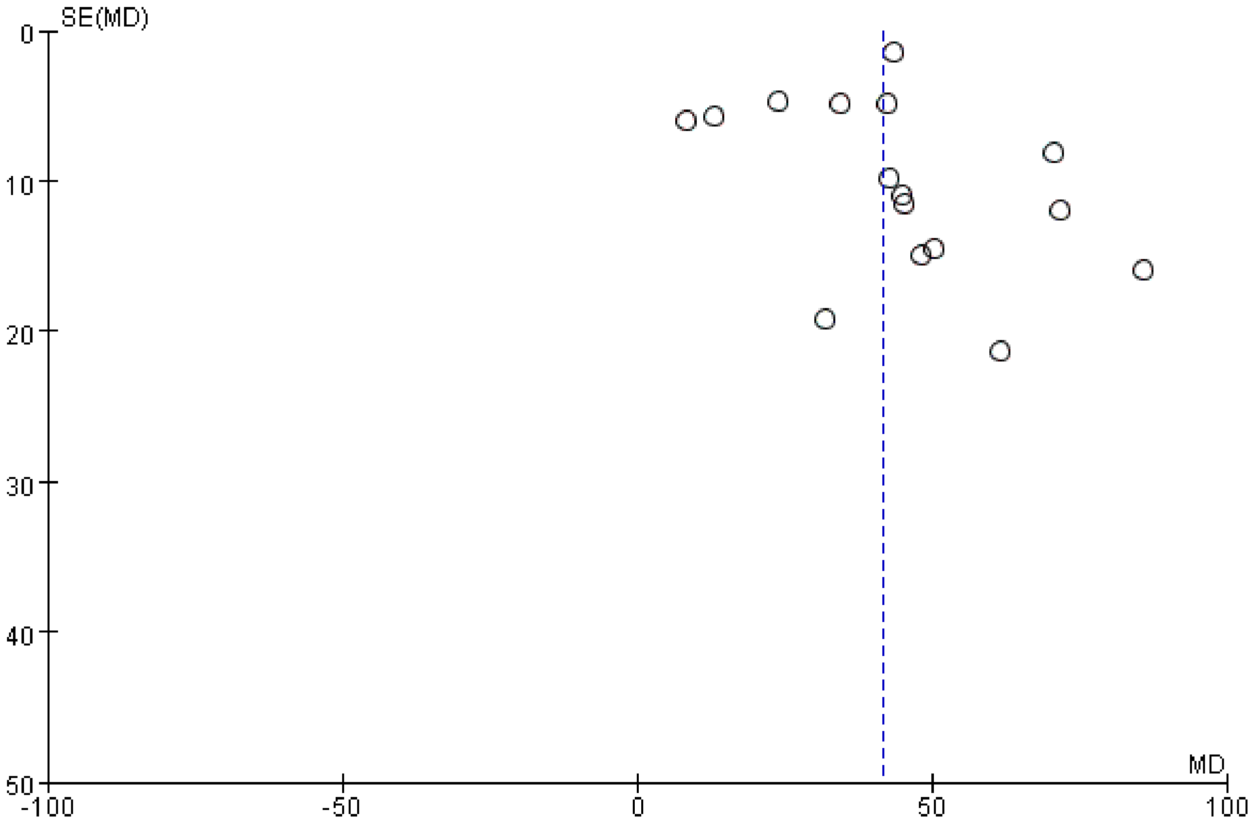

Funnel plot of comparison: 2 Cardiac function, outcome: 2.3 6-minute walk test.

Study or Subgroup	baduanjin Mean	baduanjin SD	baduanjin Total	Control Mean	Control SD	Control Total	Weight	Mean Difference IV, Random, 95% CI
**5.6.1 heart failure**								
Huimin Tang 2019	85.97	5.220733	28	42.53	5.456752	28	9.5%	43.44 [40.64, 46.24]
Lu Wang 2023	57	21.20375	40	22.72	22.43343	40	8.7%	34.28 [24.71, 43.85]
Min Wang 2023	122.3	75.13557	60	71.94	83.90173	60	4.9%	50.36 [21.86, 78.86]
Qianyu Li 2022	47.32	92.88523	44	15.63	90.09773	47	3.6%	31.69 [-5.95, 69.33]
Wan Pan 2019	242.29	51.74545	42	197.23	53.98322	42	6.0%	45.06 [22.44, 67.68]
Xiangfeng Deng 2019	52.45	23.99529	49	28.66	24.01	51	8.7%	23.79 [14.38, 33.20]
Xiaosu Ni 2022	51.38	29.63843	54	38.32	29.66214	54	8.4%	13.06 [1.88, 24.24]
Xueping Chen 2020	241.74	51.70462	46	196.91	53.50462	46	6.2%	44.83 [23.33, 66.33]
Yinli Jiao 2020	113.86	45.99737	40	71.04	42.2575	40	6.6%	42.82 [23.46, 62.18]
Yu Wang 2021	241.8	62.04732	31	156	63.85992	31	4.4%	85.80 [54.46, 117.14]
Yuemei Xu 2022	163.7	112.8108	45	102.29	87.42258	44	3.1%	61.41 [19.53, 103.29]
**Subtotal (95% CI)**			**479**			**483**	**69.9%**	**39.06 [29.59, 48.54]**
Heterogeneity: Tau² = 154.38; Chi² = 51.12, df = 10 (P < 0.00001); I² = 80%								
Test for overall effect: Z = 8.08 (P < 0.00001)								
**5.6.2 coronary heart disease**								
Wen Han 2022	98.72	24.14263	46	56.46	23.35848	46	8.6%	42.26 [32.55, 51.97]
**Subtotal (95% CI)**			**46**			**46**	**8.6%**	**42.26 [32.55, 51.97]**
Heterogeneity: Not applicable								
Test for overall effect: Z = 8.53 (P < 0.00001)								
**5.6.3 others**								
Xianghui Xiong 2016	195.7	46.75329	33	124.1	47.83848	30	5.8%	71.60 [48.20, 95.00]
Xuejiao Hong 2020	30.09	23.84447	33	21.89	24.96117	34	8.3%	8.20 [-3.49, 19.89]
Yuxuan Zhou 2021	137.67	41.26154	50	67.13	40.27538	50	7.4%	70.54 [54.56, 86.52]
**Subtotal (95% CI)**			**116**			**114**	**21.4%**	**49.54 [3.02, 96.06]**
Heterogeneity: Tau² = 1609.72; Chi² = 48.57, df = 2 (P < 0.00001); I² = 96%								
Test for overall effect: Z = 2.09 (P = 0.04)								
**Total (95% CI)**			**641**			**643**	**100.0%**	**41.50 [32.55, 50.45]**
Heterogeneity: Tau² = 216.96; Chi² = 100.89, df = 14 (P < 0.00001); I² = 86%								
Test for overall effect: Z = 9.09 (P < 0.00001)								
Test for subgroup differences: Chi² = 0.35, df = 2 (P = 0.84), I² = 0%								

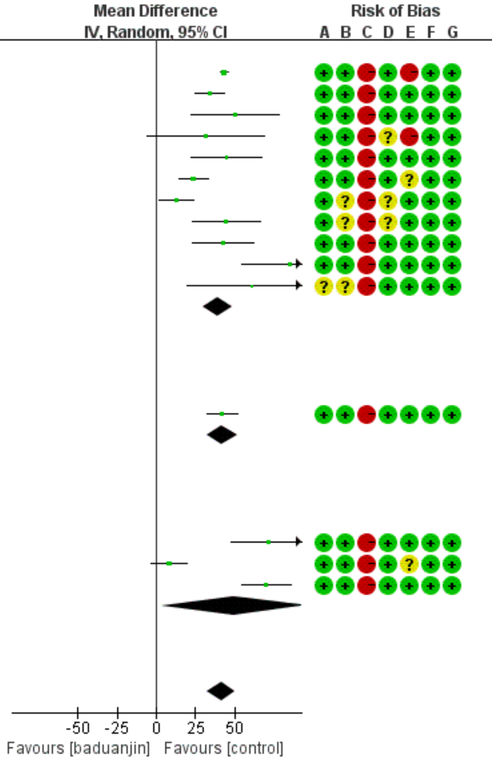

Risk of bias legend
(A) Random sequence generation (selection bias)
(B) Allocation concealment (selection bias)
(C) Blinding of participants and personnel (performance bias)
(D) Blinding of outcome assessment (detection bias)
(E) Incomplete outcome data (attrition bias)
(F) Selective reporting (reporting bias)
(G) Other bias

Forest plot of comparison: 5 Subgroups analysis, outcome: 5.6 Disease.

Study or Subgroup	baduanjin Mean	baduanjin SD	baduanjin Total	Control Mean	Control SD	Control Total	Weight	Mean Difference IV, Random, 95% CI
**5.9.1 Home-based Exercise**								
Yuxuan Zhou 2021	137.67	41.26154	50	67.13	40.27538	50	7.0%	70.54 [54.56, 86.52]
Yu Wang 2021	241.8	62.04732	31	156	63.85992	31	4.2%	85.80 [54.46, 117.14]
Xuejiao Hong 2020	30.09	23.84447	33	21.89	24.96117	34	7.9%	8.20 [-3.49, 19.89]
Xiaosu Ni 2022	51.38	29.63843	54	38.32	29.66214	54	8.0%	13.06 [1.88, 24.24]
Xianghui Xiong 2016	195.7	46.75329	33	124.1	47.83848	30	5.5%	71.60 [48.20, 95.00]
Xiangfeng Deng 2019	52.45	23.99529	49	28.66	24.01	51	8.3%	23.79 [14.38, 33.20]
Wen Han 2022	98.72	24.14263	46	56.46	23.35848	46	8.3%	42.26 [32.55, 51.97]
Shuling Fang 2022	41.9	51.85782	22	-6.25	47.49353	22	4.5%	48.15 [18.77, 77.53]
Qianyu Li 2022	47.32	92.88523	44	15.63	90.09773	47	3.4%	31.69 [-5.95, 69.33]
Min Wang 2023	122.3	75.13557	60	71.94	83.90173	60	4.6%	50.36 [21.86, 78.86]
Lu Wang 2023	57	21.20375	40	22.72	22.43343	40	8.3%	34.28 [24.71, 43.85]
**Subtotal (95% CI)**			**462**			**465**	**70.0%**	**41.32 [28.33, 54.31]**
Heterogeneity: Tau² = 378.07; Chi² = 81.72, df = 10 (P < 0.00001); I² = 88%								
Test for overall effect: Z = 6.23 (P < 0.00001)								
**5.9.2 Hospital-based Exercise**								
Yuemei Xu 2022	163.7	112.8108	45	102.29	87.42258	44	2.9%	61.41 [19.53, 103.29]
Huimin Tang 2019	85.97	5.220733	28	42.53	5.456752	28	9.1%	43.44 [40.64, 46.24]
**Subtotal (95% CI)**			**73**			**72**	**12.1%**	**43.52 [40.73, 46.31]**
Heterogeneity: Tau² = 0.00; Chi² = 0.70, df = 1 (P = 0.40); I² = 0%								
Test for overall effect: Z = 30.56 (P < 0.00001)								
**5.9.4 Others**								
Yinli Jiao 2020	113.86	45.99737	40	71.04	42.2575	40	6.3%	42.82 [23.46, 62.18]
Xueping Chen 2020	241.74	51.70462	46	196.91	53.50462	46	5.9%	44.83 [23.33, 66.33]
Wan Pan 2019	242.29	51.74545	42	197.23	53.98322	42	5.7%	45.06 [22.44, 67.68]
**Subtotal (95% CI)**			**128**			**128**	**17.9%**	**44.11 [31.97, 56.24]**
Heterogeneity: Tau² = 0.00; Chi² = 0.03, df = 2 (P = 0.99); I² = 0%								
Test for overall effect: Z = 7.12 (P < 0.00001)								
**Total (95% CI)**			**663**			**665**	**100.0%**	**41.75 [33.08, 50.42]**

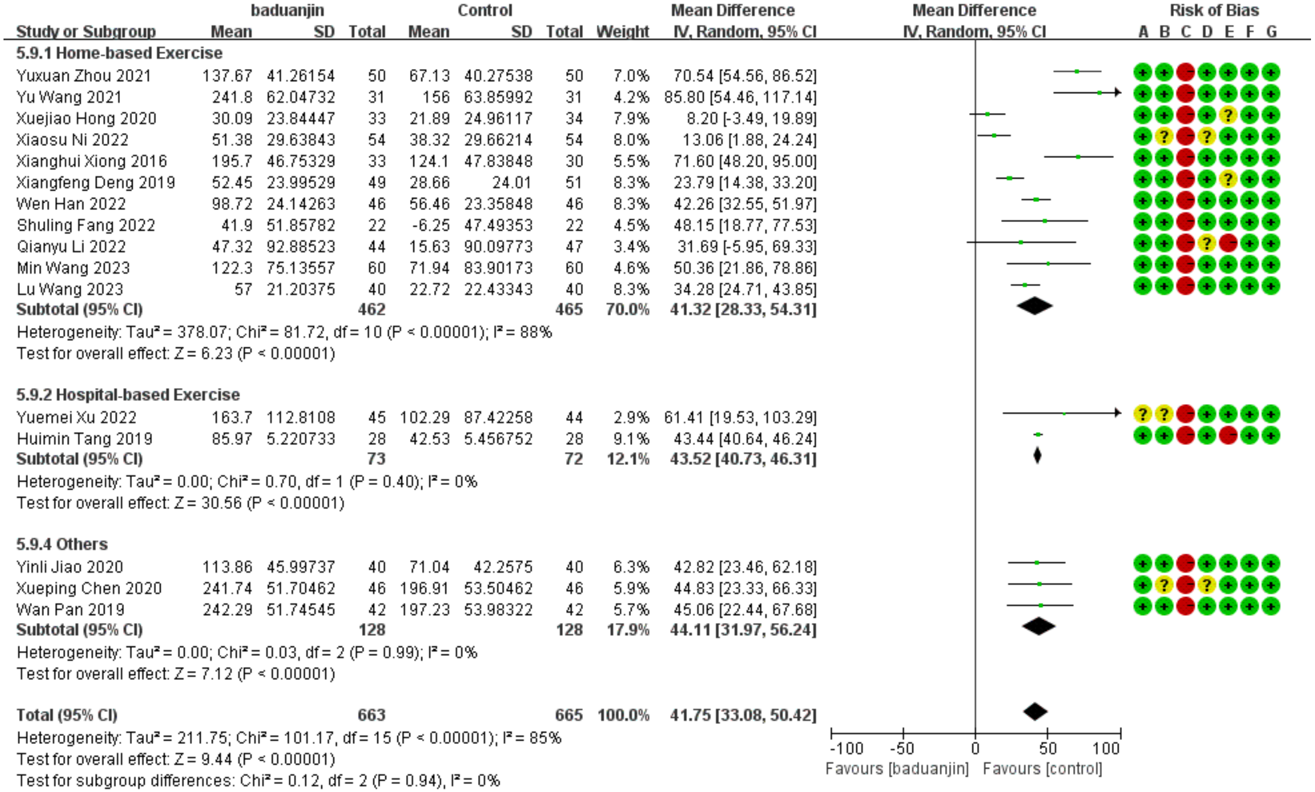

Heterogeneity: Tau² = 211.75; Chi² = 101.17, df = 15 (P < 0.00001); I² = 85%
Test for overall effect: Z = 9.44 (P < 0.00001)
Test for subgroup differences: Chi² = 0.12, df = 2 (P = 0.94), I² = 0%

Risk of bias legend
(A) Random sequence generation (selection bias)
(B) Allocation concealment (selection bias)
(C) Blinding of participants and personnel (performance bias)
(D) Blinding of outcome assessment (detection bias)
(E) Incomplete outcome data (attrition bias)
(F) Selective reporting (reporting bias)
(G) Other bias

Forest plot of comparison: 5 Subgroups analysis, outcome: 5.9 Exercise Facility.

	Baduanjin			Control				Mean Difference
**Study or Subgroup**	**Mean**	**SD**	**Total**	**Mean**	**SD**	**Total**	**Weight**	**IV, Random, 95% CI**
Canjie Hong 2022	12.7	5.160504	60	8.05	4.991923	60	7.6%	4.65 [2.83, 6.47]
Huiqi Wang 2018	4.87	1.931243	32	1	3.228142	32	7.9%	3.87 [2.57, 5.17]
Junhua Ke 2020	4.8	3.686462	30	1.1	3.487119	30	7.6%	3.70 [1.88, 5.52]
Lei Zhang 2022	3	31.19295	40	1	3.605551	40	2.3%	2.00 [-7.73, 11.73]
Lu Wang 2023	8	3.916593	40	3.19	3.613267	40	7.7%	4.81 [3.16, 6.46]
Min Wang 2023	6.23	4.652236	60	3.47	4.988667	60	7.6%	2.76 [1.03, 4.49]
Wan Pan 2019	8.71	5.498609	42	2.39	5.61319	42	7.1%	6.32 [3.94, 8.70]
Wen Han 2022	19.58	6.225496	46	6.3	5.846871	46	7.1%	13.28 [10.81, 15.75]
Xiangfeng Deng 2019	0.59	1.992712	49	0.35	2.461727	51	8.1%	0.24 [-0.64, 1.12]
Xianghui Xiong 2016	8.7	5.566866	33	3.2	5.406478	30	6.9%	5.50 [2.79, 8.21]
Xuejiao Hong 2020	6.79	7.871906	33	5.59	7.207628	34	6.1%	1.20 [-2.42, 4.82]
Yanan Wang 2022	8.98	10.04128	45	6.51	10.16515	45	5.6%	2.47 [-1.70, 6.64]
Yuemei Xu 2022	1.41	9.447015	45	1.7	8.261955	44	6.0%	-0.29 [-3.97, 3.39]
Yuxuan Zhou 2021	9.99	11.73769	50	1.85	14.55397	50	4.7%	8.14 [2.96, 13.32]
Zibo Shi 2018	1	2.645751	26	1	3	29	7.8%	0.00 [-1.49, 1.49]
**Total (95% CI)**			**631**			**633**	**100.0%**	**3.93 [2.21, 5.65]**

Heterogeneity: Tau² = 9.34; Chi² = 148.59, df = 14 (P < 0.00001); I² = 91%

Test for overall effect: Z = 4.47 (P < 0.00001)

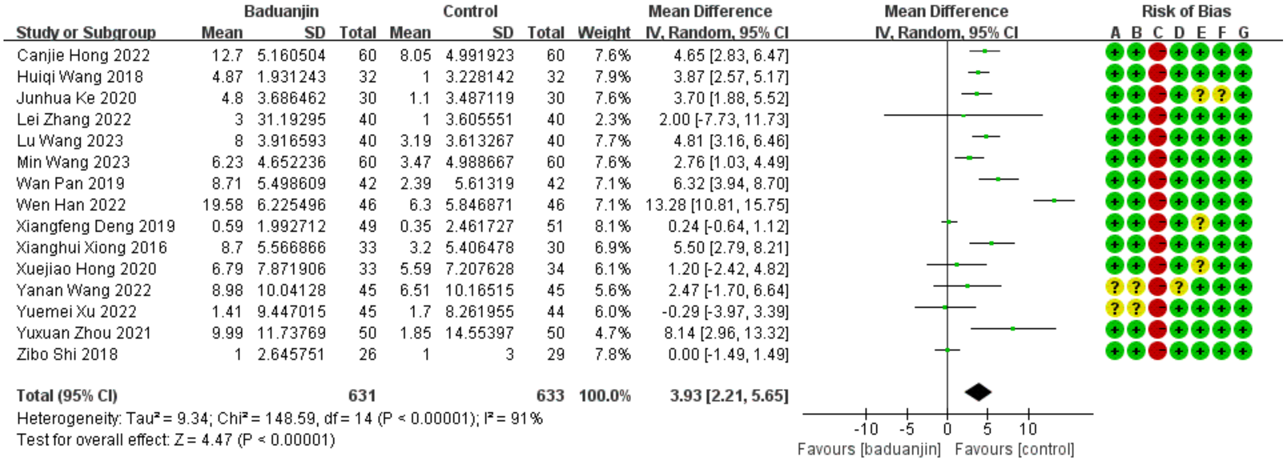

Risk of bias legend

(A) Random sequence generation (selection bias)

(B) Allocation concealment (selection bias)

(C) Blinding of participants and personnel (performance bias)

(D) Blinding of outcome assessment (detection bias)

(E) Incomplete outcome data (attrition bias)

(F) Selective reporting (reporting bias)

(G) Other bias

Forest plot of comparison: 2 Cardiac function, outcome: 2.8 LVEF.

2.6LVESD

Study or Subgroup	Baduanjin Mean	Baduanjin SD	Baduanjin Total	Control Mean	Control SD	Control Total	Weight	Mean Difference IV, Random, 95% CI	A	B	C	D	E	F	G
Wei Qi 2020	-4.62	1.0006	50	-3.3	0.597746	50	27.7%	-1.32 [-1.64, -1.00]	?	?	-	+	+	+	+
Wen Han 2022	-11.17	3.803538	46	-7.29	3.849364	46	17.9%	-3.88 [-5.44, -2.32]	+	+	-	+	+	+	+
Yanan Wang 2022	-4.21	0.923688	45	-2.04	1.387516	45	26.8%	-2.17 [-2.66, -1.68]	?	?	-	?	+	+	+
Yuxuan Zhou 2021	-1.67	1.046757	50	-1.55	0.639296	50	27.6%	-0.12 [-0.46, 0.22]	+	+	-	+	+	+	+
**Total (95% CI)**			**191**			**191**	**100.0%**	**-1.67 [-2.76, -0.59]**							

Heterogeneity: Tau² = 1.08; Chi² = 64.30, df = 3 (P < 0.00001); I² = 95%

Test for overall effect: Z = 3.02 (P = 0.002)

Mean Difference IV, Random, 95% CI

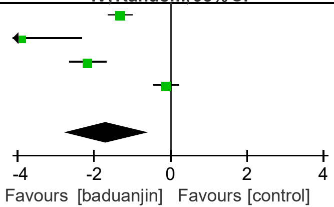

Risk of Bias

Risk of bias legend

(**A**) Random sequence generation (selection bias)

(**B**) Allocation concealment (selection bias)

(**C**) Blinding of participants and personnel (performance bias)

(**D**) Blinding of outcome assessment (detection bias)

(**E**) Incomplete outcome data (attrition bias)

(**F**) Selective reporting (reporting bias)

(**G**) Other bias

2.7 LVDD

Study or Subgroup	Baduanjin Mean	Baduanjin SD	Baduanjin Total	Control Mean	Control SD	Control Total	Weight	Mean Difference IV, Random, 95% CI	A	B	C	D	E	F	G
Huiqi Wang 2018	-2.1	3.87	32	-1.37	2.838679	32	16.6%	-0.73 [-2.39, 0.93]	+	+	−	+	+	+	+
Wei Qi 2020	-3.97	1.297228	50	-3	1.158792	50	23.3%	-0.97 [-1.45, -0.49]	?	?	−	+	+	+	+
Wen Han 2022	-25.54	5.215007	46	-18.03	5.368696	46	13.7%	-7.51 [-9.67, -5.35]	+	+	−	+	+	+	+
Yanan Wang 2022	-3.9	1.225887	45	-1	1.515619	45	23.0%	-2.90 [-3.47, -2.33]	?	?	−	?	+	+	+
Yuxuan Zhou 2021	-3.87	1.218647	50	-2.41	1.17911	50	23.4%	-1.46 [-1.93, -0.99]	+	+	−	+	+	+	+
**Total (95% CI)**			**223**			**223**	**100.0%**	**-2.38 [-3.59, -1.17]**							

Heterogeneity: Tau² = 1.57; Chi² = 55.85, df = 4 (P < 0.00001); I² = 93%
Test for overall effect: Z = 3.86 (P = 0.0001)

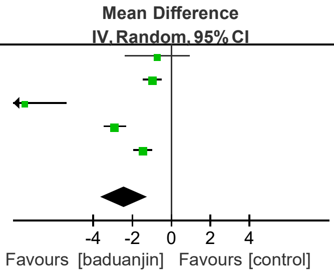

Risk of bias legend
(**A**) Random sequence generation (selection bias)
(**B**) Allocation concealment (selection bias)
(**C**) Blinding of participants and personnel (performance bias)
(**D**) Blinding of outcome assessment (detection bias)
(**E**) Incomplete outcome data (attrition bias)
(**F**) Selective reporting (reporting bias)
(**G**) Other bias

2.4NT-proBNP

Study or Subgroup	Baduanjin Mean	Baduanjin SD	Baduanjin Total	Control Mean	Control SD	Control Total	Weight	Mean Difference IV, Random, 95% CI	Risk of Bias A	B	C	D	E	F	G
Lei Zhang 2022	-1,103.82	283.108	40	-915.65	328.7465	40	13.9%	-188.17 [-322.62, -53.72]	+	+	-	+	+	+	+
Min Wang 2023	-1,786.98	1,988.311	60	-674.93	2,021.729	60	2.6%	-1112.05 [-1829.55, -394.55]	+	+	-	+	+	+	+
Wan Pan 2019	-3,303.92	751.7001	42	-3,001.95	780.4209	42	7.8%	-301.97 [-629.67, 25.73]	+	+	-	+	+	+	+
Xianghui Xiong 2016	-3,302.9	782.5827	33	-3,182.9	796.6796	30	6.4%	-120.00 [-510.60, 270.60]	+	+	-	+	+	+	+
Xiaosu Ni 2022	-707.88	193.6159	54	-639.95	200.2577	54	15.6%	-67.93 [-142.22, 6.36]	+	?	-	?	+	+	+
Xuejiao Hong 2020	-339.73	260.8767	33	-286.62	241.0719	34	14.3%	-53.11 [-173.48, 67.26]	+	+	-	+	?	+	+
Yinli Jiao 2020	-351.83	229.4084	40	-344.57	241.2834	40	14.8%	-7.26 [-110.44, 95.92]	+	+	-	+	+	+	+
Yu Wang 2021	-1,454.7	498.1266	31	-745.1	396.3607	31	10.8%	-709.60 [-933.69, -485.51]	+	+	-	+	+	+	+
Zibo Shi 2018	-714.39	273.5175	26	-702.1	224.5938	29	13.9%	-12.29 [-145.46, 120.88]	+	+	-	+	+	+	+
**Total (95% CI)**			**359**			**360**	**100.0%**	**-183.83 [-309.83, -57.83]**							

Heterogeneity: Tau² = 25125.85; Chi² = 45.24, df = 8 (P < 0.00001); I² = 82%
Test for overall effect: Z = 2.86 (P = 0.004)

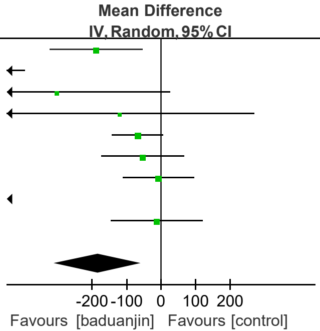

Risk of bias legend
(**A**) Random sequence generation (selection bias)
(**B**) Allocation concealment (selection bias)
(**C**) Blinding of participants and personnel (performance bias)
(**D**) Blinding of outcome assessment (detection bias)
(**E**) Incomplete outcome data (attrition bias)
(**F**) Selective reporting (reporting bias)
(**G**) Other bias

3.1 serum NO

Study or Subgroup	Experimental Mean	Experimental SD	Experimental Total	Control Mean	Control SD	Control Total	Weight	Mean Difference IV, Random, 95% CI	A	B	C	D	E	F	G
Chunmei Xiao 2016	3.3	4.70319	24	-0.3	4.7	24	45.9%	3.60 [0.94, 6.26]	+	+	−	+	+	+	+
Liwei Zheng 2014	3.37	4.74564	27	-0.12	4.510133	28	54.1%	3.49 [1.04, 5.94]	+	−	−	+	+	+	+
**Total (95% CI)**			**51**			**52**	**100.0%**	**3.54 [1.74, 5.34]**							

Heterogeneity: Tau² = 0.00; Chi² = 0.00, df = 1 (P = 0.95); I² = 0%
Test for overall effect: Z = 3.85 (P = 0.0001)

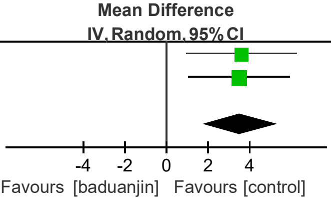

Risk of bias legend
(**A**) Random sequence generation (selection bias)
(**B**) Allocation concealment (selection bias)
(**C**) Blinding of participants and personnel (performance bias)
(**D**) Blinding of outcome assessment (detection bias)
(**E**) Incomplete outcome data (attrition bias)
(**F**) Selective reporting (reporting bias)
(**G**) Other bias

4.4MLHFQ

Study or Subgroup	baduanjin Mean	baduanjin SD	baduanjin Total	Control Mean	Control SD	Control Total	Weight	Mean Difference IV, Random, 95% CI	A	B	C	D	E	F	G
Huimin Tang 2019	-14.82	3.670128	28	-8.57	4.144406	28	19.2%	-6.25 [-8.30, -4.20]	+	+	−	+	−	+	+
Xuejiao Hong 2020	-5.52	7.924323	33	-3.15	6.488058	34	15.6%	-2.37 [-5.84, 1.10]	+	+	−	+	?	+	+
Yinli Jiao 2020	-20.44	8.954373	40	-10.51	6.276408	40	15.8%	-9.93 [-13.32, -6.54]	+	+	−	+	+	+	+
Yu Wang 2021	-17.3	5.350701	31	-7.6	5.702631	31	17.4%	-9.70 [-12.45, -6.95]	+	+	−	+	+	+	+
Yuemei Xu 2022	-34.81	6.351559	45	-30.93	4.588605	44	18.6%	-3.88 [-6.18, -1.58]	?	?	−	+	+	+	+
Zibo Shi 2018	-17.92	8.492638	26	-7.14	7.816259	29	13.4%	-10.78 [-15.11, -6.45]	+	+	−	+	+	+	+
**Total (95% CI)**			**203**			**206**	**100.0%**	**-7.00 [-9.54, -4.45]**							

Heterogeneity: $Tau^2 = 7.70$; $Chi^2 = 23.35$, df = 5 ($P = 0.0003$); $I^2 = 79\%$

Test for overall effect: $Z = 5.38$ ($P < 0.00001$)

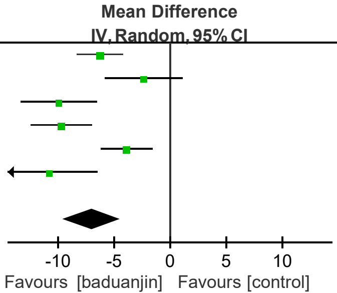

Risk of bias legend

(**A**) Random sequence generation (selection bias)

(**B**) Allocation concealment (selection bias)

(**C**) Blinding of participants and personnel (performance bias)

(**D**) Blinding of outcome assessment (detection bias)

(**E**) Incomplete outcome data (attrition bias)

(**F**) Selective reporting (reporting bias)

(**G**) Other bias

Study	Sample Size (Dropout rate)	Sex	Age or Age range （average）	Type of patients	Intervention group	Control grop	Treatment or Exercise Programme	Adverse effects report	Outcome
**Wang2018**	64(0%)<br>BJ 32/32(100%)<br>CG: 32/32(100%)	M:34<br>F:30	29~72<br>(56.8±12.3)	Patients recovering from coronary heart disease	32	32	40min/day 、5days/week	NO	LA、LVEDD、LVEF、E / A、VC -FEV1-MV
**Qi2020**	100(0%)<br>BJ: 50/50(100%)<br>CG: 50/50(100%)	BJ: M:34 F:16<br>CG: M:37 F:13<br>T: M:71 F:29	BJ: (62.8 + 9.7)<br>CG: (65.2 ± 7.8)	Patients with chronic heart failure	50	50	Step-up training	NO	LVEDD、LVESD、LVEF、MLHFQ score (Quality of life score)
**Wang、Lei、Zhang2023**	120(0%)<br>BJ: 60/60(100%)<br>CG: 60/60(100%)	T: M:67 F:53<br>BJ: M:33 F:27<br>CG: M:34 F:26	Z:41~85 (62.55 ±10.78)<br>BJ:42~85 (65.18 ±9. 92<br>CG:41~79 (59.92 ± 11. 03	Patients with chronic heart failure	60	60	30min/day、6days/week	NO	Cardiac function classification 、NYHA grade、NT-proBNP、6MWT 、 SV 、CO、 CI、LVEF、Cardiac function index

**Jiao 、Wu2020**	80(0%)<br>BJ:<br>40/40(100%)<br>CG:<br>40/40(100%)	BJ:<br>M:18<br>F:22<br>CG:<br>M:19<br>F:21<br>T:<br>M:37<br>F:43	BJ: 57 ~ 74 （66.6±6.32）<br>CG: 59 ~ 75 （64.32±5.23）	Patients with chronic heart failure	40	40	10min/time、<br>Once in the morning and once in the evening<br>6month		NT-pro BNP、<br>LVEF、<br>6MWT、<br>MLHFQ
**Zhou 、Li2021**	100(0%)<br>BJ:<br>50/50(100%)<br>CG:<br>50/50(100%)	BJ:<br>M:27<br>F:23<br>CG:<br>M:31<br>F:19<br>T:<br>M:58<br>F:42	BJ: 51~66 (57.87± 4.61)<br>CG: 52~68 (58.41±4.52)	Patients with cardiac function after myocardial infarction were classified as grade I or grade II	50	50	Exercise once in the morning and once in the afternoon for 30 minutes each time.<br>3month	NO	6MWT、<br>Quality of life score、LVESD、<br>LVEDD、LVEF
**Zhang2022**	80(0%)<br>BJ:<br>40/40(100%)<br>CG:<br>40/40(100%)	BJ:<br>M:24<br>F:16<br>CG:<br>M:22<br>F:18<br>T:	BJ: （56±11）<br>CG: （54±13）	Patients with chronic heart failure	40	40	1 ~ 2 time/day、<br>Every time 15 ~ 40 min、<br>12week	NO	LVEF 、<br>Mental health score（MCS）、<br>NT-ProBNP

		M:46<br>F:34							
**Ke2020**	60(0%)<br>BJ:<br>30/30(100%)<br>CG:<br>30/30(100%)	BJ:<br>M:21<br>F:9<br>CG:<br>M:16<br>F:14<br>T:<br>M:37<br>F:23	BJ(65.33)<br>CG(67.80)	Patients with chronic heart failure	30	30	5time/week、<br>Every time60 min、<br>24week	NO	LVEF、 LVESV 、LVEDV、<br>NE、TNF-a、<br>IL-1、IL-6
**Wang 、 Pan2021**	93（0%）<br>BJ31/31(100%)<br>CG31/31(100%)	BJ:<br>M: 16<br>F： 15<br>CG:<br>M: 19<br>F： 12<br>T:<br>M: 35<br>F： 27	BJ(60.5±8.0)<br>CG（62.1±9.1）	Elderly patients with chronic heart failure	31	31	2 time/ d、<br>2 group/ time、<br>Every group 8 ～ 10 min、<br>180 day	NO	MLHFQ score、<br>NYHA grade 、<br>TCM syndrome points、<br>NT-proBNP 、<br>6MWT
**Zhang2022**	80(0%)<br>BJ:<br>40/40(100%)<br>CG:<br>40/40(100%)	BJ:<br>M: 25<br>F： 15<br>CG:<br>M: 22	BJ<br>67.98±4.87<br>CG<br>67.14±5.01	Elderly hypertensive patients	40	40	5d/week、1 time/ day、<br>Every time 30-40min<br>6 month	NO	SBP 、 DBP、<br>SAS score 、<br>SDS score、<br>PSQI score

		F：18<br>T:<br>M：47<br>F：33							
**Fan、Yao、Fan2022**	47(94%)<br>BJ:<br>22/24(92%)<br>CG:<br>22/23(96%)	BJ:<br>M：9<br>F：13<br>CG:<br>M：13<br>F：9<br>T:<br>M：22<br>F：22	BJ<br>6 1— 7 9<br>(72.14±4.78)<br>CG<br>66— 8 1<br>(74.50±4.02)	Elderly patients with heart failure and asthenia	22	22	12week<br>5time/week 、<br>35min/time	No	Quality of life score （MLHFQ）、6MWT 、BNP 、asthenia
**Shan2015**	130（0%)<br>BJ:<br>55/55(100%)<br>CG:<br>20/20(100%)	M：130	20~25	without	55	20	60min/day、<br>5time/week<br>4week	No	SV、CL、VPE、HR、MSP、MDP、TR、AC、
**Zheng 、Chen2014**	55/60(92%)<br>BJ:<br>27/30(90%)<br>CG:<br>28/30(93%)	BJ:<br>M：13<br>F：14<br>CG:<br>M：16<br>F：12<br>T:<br>M：29	BJ:<br>69.23±3.72<br>CG:<br>70.06±3.51	Elderly patients with grade I hypertension	27	28	5d/week、<br>1time/day 、<br>Everytime30min、<br>12week	No	SBP、 DBP、NO、ET-1

		F：26							
**Qin2012**	30（0%）<br>BJ:<br>10/10(100%)<br>CG:<br>10/10(100%)	not	21~22	without	10	10	20week、<br>5time /week、<br>Every time 60min	No	HR、SV、SI、CI、VPE 、 MSP 、MDP、MAP、TR
**Wang2022**	90（0%）<br>BJ:<br>45/45(100%)<br>CG:<br>45/45(100%)	BJ:<br>M：21<br>F：24<br>CG:<br>M：23<br>F：22<br>T:<br>M：44<br>F：46	BJ:<br>45~72<br>（53.58±7.12）<br>CG:<br>43~68<br>（52.36±6.78）	Patients with chronic heart failure	45	45	1time/day、<br>Everytime30min、<br>5d~7d/week、<br>2 month	NO	MLHFQ、<br>Quality of life score（Physical limitations 、 emotions 、 symptoms 、 social limitations<br>LVES、 LVEDD、LVEF
**Ni2022**	108（0%）<br>BJ:<br>54/54(100%)<br>CG:<br>54/54(100%)	BJ:<br>M：30<br>F：24<br>CG:<br>M：29<br>F：25<br>T:<br>M59	BJ:<br>48~79<br>(61. 26 ±3. 24)<br>CG:<br>47~80<br>(61.33 ± 3.51)	Patients with chronic heart failure	54	54	Every time 15min、<br>2time/day<br>3month	NO	Mental resilience (EHFSCBS) 、 MLHFQ 、 6MWT 、 NT - proBNP

		F：49							
**Xiao2016**	48（0%）<br>BJ:<br>24/24(100%)<br>CG:<br>24/24(100%)	not	(65.6±7.8)	Essential hypertensive patient	24	24	30min/day、5time/week 6month	No	SBP、DBP、HDL-C、LDL-C、TC、Triglycerides 、blood sugar 、NO、ET-1

BJ: baduanjin、 CG: control group; M:male、 F:female；T:total

PRISMA 2020 Checklist

Section and Topic	Item #	Checklist item	Location where item is reported
**TITLE**			
Title	1	Identify the report as a systematic review.	Title
**ABSTRACT**			
Abstract	2	See the PRISMA 2020 for Abstracts checklist.	Abstract
**INTRODUCTION**			
Rationale	3	Describe the rationale for the review in the context of existing knowledge.	Introduction
Objectives	4	Provide an explicit statement of the objective(s) or question(s) the review addresses.	Introduction
**METHODS**			
Eligibility criteria	5	Specify the inclusion and exclusion criteria for the review and how studies were grouped for the syntheses.	Selection and Exclusion Criteria
Information sources	6	Specify all databases, registers, websites, organisations, reference lists and other sources searched or consulted to identify studies. Specify the date when each source was last searched or consulted.	Search strategy
Search strategy	7	Present the full search strategies for all databases, registers and websites, including any filters and limits used.	Supplementary Methods S1
Selection process	8	Specify the methods used to decide whether a study met the inclusion criteria of the review, including how many reviewers screened each record and each report retrieved, whether they worked independently, and if applicable, details of automation tools used in the process.	Study Identification and Selection
Data collection process	9	Specify the methods used to collect data from reports, including how many reviewers collected data from each report, whether they worked independently, any processes for obtaining or confirming data from study investigators, and if applicable, details of automation tools used in the process.	Data Extraction and Quality Assessment
Data items	10a	List and define all outcomes for which data were sought. Specify whether all results that were compatible with each outcome domain in each study were sought (e.g. for all measures, time points, analyses), and if not, the methods used to decide which results to collect.	Outcomes
	10b	List and define all other variables for which data were sought (e.g. participant and intervention characteristics, funding sources). Describe any assumptions made about any missing or unclear information.	Outcomes
Study risk of bias assessment	11	Specify the methods used to assess risk of bias in the included studies, including details of the tool(s) used, how many reviewers assessed each study and whether they worked independently, and if applicable, details of automation tools used in the process.	Data Extraction and Quality Assessment
Effect measures	12	Specify for each outcome the effect measure(s) (e.g. risk ratio, mean difference) used in the synthesis or presentation of results.	Statistical Analysis
Synthesis methods	13a	Describe the processes used to decide which studies were eligible for each synthesis (e.g. tabulating the study intervention characteristics and comparing against the planned groups for each synthesis (item #5)).	Statistical Analysis
	13b	Describe any methods required to prepare the data for presentation or synthesis, such as handling of missing summary statistics, or data conversions.	Statistical Analysis
	13c	Describe any methods used to tabulate or visually display results of individual studies and syntheses.	Statistical Analysis
	13d	Describe any methods used to synthesize results and provide a rationale for the choice(s). If meta-analysis was performed, describe the model(s), method(s) to identify the presence and extent of statistical heterogeneity, and software package(s) used.	Statistical Analysis

Section and Topic	Item #	Checklist item	Location where item is reported
	13e	Describe any methods used to explore possible causes of heterogeneity among study results (e.g. subgroup analysis, meta-regression).	Statistical Analysis
	13f	Describe any sensitivity analyses conducted to assess robustness of the synthesized results.	Statistical Analysis
Reporting bias assessment	14	Describe any methods used to assess risk of bias due to missing results in a synthesis (arising from reporting biases).	Quality Assessment of Included Studies
Certainty assessment	15	Describe any methods used to assess certainty (or confidence) in the body of evidence for an outcome.	Quality Assessment of Included Studies

Section and Topic	Item #	Checklist item	Location where item is reported
**RESULTS**			
Study selection	16a	Describe the results of the search and selection process, from the number of records identified in the search to the number of studies included in the review, ideally using a flow diagram.	Study Identification and Selection
	16b	Cite studies that might appear to meet the inclusion criteria, but which were excluded, and explain why they were excluded.	Study Identification and Selection
Study characteristics	17	Cite each included study and present its characteristics.	Characteristics of the Included Studies
Risk of bias in studies	18	Present assessments of risk of bias for each included study.	Quality Assessment of Included Studies
Results of individual studies	19	For all outcomes, present, for each study: (a) summary statistics for each group (where appropriate) and (b) an effect estimate and its precision (e.g. confidence/credible interval), ideally using structured tables or plots.	Analysis of Outcomes
Results of syntheses	20a	For each synthesis, briefly summarise the characteristics and risk of bias among contributing studies.	Analysis of Outcomes
	20b	Present results of all statistical syntheses conducted. If meta-analysis was done, present for each the summary estimate and its precision (e.g. confidence/credible interval) and measures of statistical heterogeneity. If comparing groups, describe the direction of the effect.	Analysis of Outcomes
	20c	Present results of all investigations of possible causes of heterogeneity among study results.	Analysis of Outcomes
	20d	Present results of all sensitivity analyses conducted to assess the robustness of the synthesized results.	Analysis of Outcomes
Reporting biases	21	Present assessments of risk of bias due to missing results (arising from reporting biases) for each synthesis assessed.	Supplementary Figure S6
Certainty of evidence	22	Present assessments of certainty (or confidence) in the body of evidence for each outcome assessed.	Table 3
**DISCUSSION**			

Discussion	23a	Provide a general interpretation of the results in the context of other evidence.	Summary of Findings
	23b	Discuss any limitations of the evidence included in the review.	Limitations
	23c	Discuss any limitations of the review processes used.	Limitations
	23d	Discuss implications of the results for practice, policy, and future research.	Implications for Clinical Practice
**OTHER INFORMATION**			
Registration and protocol	24a	Provide registration information for the review, including register name and registration number, or state that the review was not registered.	Abstract
	24b	Indicate where the review protocol can be accessed, or state that a protocol was not prepared.	Abstract
	24c	Describe and explain any amendments to information provided at registration or in the protocol.	Abstract
Support	25	Describe sources of financial or non-financial support for the review, and the role of the funders or sponsors in the review.	Sponsor's Role
Competing interests	26	Declare any competing interests of review authors.	Conflict of Interest
Availability of data, code and other materials	27	Report which of the following are publicly available and where they can be found: template data collection forms; data extracted from included studies; data used for all analyses; analytic code; any other materials used in the review.	Acknowledgm ents

From: Page MJ, McKenzie JE, Bossuyt PM, Boutron I, Hoffmann TC, Mulrow CD, et al. The PRISMA 2020 statement: an updated guideline for reporting systematic reviews. BMJ 2021;372:n71. doi: 10.1136/bmj.n71

Author(s):
Date: 2023-11-15
Question: age for cardiovascular disease
Settings:
Bibliography: . BDJ exercise for cardiovascular disease. Cochrane Database of Systematic Reviews [Year], Issue [Issue].

Quality assessment							No of patients		Effect		Quality	Importance
No of studies	Design	Risk of bias	Inconsistency	Indirectness	Imprecision	Other considerations	Age	Control	Relative (95% CI)	Absolute		
**male**												
24	randomised trials	serious[1]	no serious inconsistency	serious[1]	no serious imprecision	none	551/992 (55.5%)	580/998 (58.1%)	OR 0.9 (0.75 to 1.08)	26 fewer per 1000 (from 71 fewer to 19 more)	⊕⊕OO LOW	NOT IMPORTANT
								56.7%		26 fewer per 1000 (from 72 fewer to 19 more)		
**female**												
23	randomised trials	serious[1]	no serious inconsistency	serious[1]	no serious imprecision	none	417/952 (43.8%)	397/958 (41.4%)	OR 1.1 (0.92 to 1.32)	23 more per 1000 (from 20 fewer to 69 more)	⊕⊕OO LOW	NOT IMPORTANT
								43.2%		24 more per 1000 (from 20 fewer to 69 more)		
**age (Better indicated by lower values)**												
22	randomised trials	serious[1]	no serious inconsistency	no serious indirectness	no serious imprecision	none	888	892	-	MD 0.72 higher (0.26 to 1.17 higher)	⊕⊕⊕O MODERATE	NOT IMPORTANT

[1] No explanation was provided

Author(s):
Date: 2023-11-15
Question: Cardiac function for cardiovascular disease
Settings:
Bibliography: . BDJ exercise for cardiovascular disease. Cochrane Database of Systematic Reviews [Year], Issue [Issue].

Quality assessment							No of patients		Effect		Quality	Importance
No of studies	Design	Risk of bias	Inconsistency	Indirectness	Imprecision	Other considerations	Cardiac function	Control	Relative (95% CI)	Absolute		
**DBP (Better indicated by lower values)**												
3	randomised trials	no serious risk of bias[1]	no serious inconsistency	no serious indirectness	no serious imprecision	none	91	92	-	MD 7.32 lower (15.44 lower to 0.8 higher)	⊕⊕⊕⊕ HIGH	IMPORTANT
**SBP (Better indicated by lower values)**												
3	randomised trials	no serious risk of bias	no serious inconsistency	no serious indirectness	no serious imprecision	none	91	92	-	MD 9.56 lower (17.23 to 1.89 lower)	⊕⊕⊕⊕ HIGH	IMPORTANT
**6-minute walk test (Better indicated by lower values)**												
16	randomised trials	serious[1]	no serious inconsistency	no serious indirectness	no serious imprecision	none	663	665	-	MD 41.75 higher (33.08 to 50.42 higher)	⊕⊕⊕O MODERATE	CRITICAL
**NT-proBNP (Better indicated by lower values)**												
9	randomised trials	no serious risk of bias	no serious inconsistency	no serious indirectness	no serious imprecision	none	359	360	-	MD 183.83 lower (309.83 to 57.83 lower)	⊕⊕⊕⊕ HIGH	CRITICAL
**SV (Better indicated by lower values)**												
2	randomised trials	no serious risk of bias	no serious inconsistency	no serious indirectness	no serious imprecision	none	120	120	-	MD 3.36 higher (1.34 lower to 8.05 higher)	⊕⊕⊕⊕ HIGH	IMPORTANT
**LVESD (Better indicated by lower values)**												
4	randomised trials	serious[1]	no serious inconsistency	no serious indirectness	no serious imprecision	none	191	191	-	MD 1.67 lower (2.76 to	⊕⊕⊕O MODERATE	IMPORTANT

										0.59 lower)		
**LVDD (Better indicated by lower values)**												
5	randomised trials	serious[1]	no serious inconsistency	no serious indirectness	no serious imprecision	none	223	223	-	MD 2.38 lower (3.59 to 1.17 lower)	⊕⊕⊕O MODERATE	IMPORTANT
**LVEF (Better indicated by lower values)**												
15	randomised trials	serious[1]	no serious inconsistency	no serious indirectness	no serious imprecision	none	631	633	-	MD 3.93 higher (2.21 to 5.65 higher)	⊕⊕⊕O MODERATE	CRITICAL
**CI (Better indicated by lower values)**												
2	randomised trials	no serious risk of bias	no serious inconsistency	no serious indirectness	no serious imprecision	none	120	120	-	MD 0.18 higher (0.02 lower to 0.37 higher)	⊕⊕⊕⊕ HIGH	IMPORTANT

[1] No explanation was provided

Author(s):
Date: 2023-11-15
Question: Blood index for cardiovascular disease
Settings:
Bibliography: . BDJ exercise for cardiovascular disease. Cochrane Database of Systematic Reviews [Year], Issue [Issue].

Quality assessment							No of patients		Effect		Quality	Importance
No of studies	Design	Risk of bias	Inconsistency	Indirectness	Imprecision	Other considerations	Blood index	Control	Relative (95% CI)	Absolute		
**serum NO (Better indicated by lower values)**												
2	randomised trials	serious[1]	no serious inconsistency	no serious indirectness	no serious imprecision	none	51	52	-	MD 3.54 higher (1.74 to 5.34 higher)	⊕⊕⊕O MODERATE	IMPORTANT
**ET-1 (Better indicated by lower values)**												
2	randomised trials	serious[1]	no serious inconsistency	no serious indirectness	no serious imprecision	none	51	52	-	MD 9.55 lower (13.51 to 5.59 lower)	⊕⊕⊕O MODERATE	IMPORTANT

[1] No explanation was provided

Author(s):
Date: 2023-11-15
Question: Others for cardiovascular disease
Settings:
Bibliography: . BDJ exercise for cardiovascular disease. Cochrane Database of Systematic Reviews [Year], Issue [Issue].

Quality assessment							No of patients		Effect		Quality	Importance
No of studies	Design	Risk of bias	Inconsistency	Indirectness	Imprecision	Other considerations	Others	Control	Relative (95% CI)	Absolute		
**Physical limitations (Better indicated by lower values)**												
2	randomised trials	no serious risk of bias	no serious inconsistency	no serious indirectness	no serious imprecision	none	100	100	-	MD 0.79 lower (1.46 to 0.11 lower)	⊕⊕⊕⊕ HIGH	IMPORTANT
**Sentiments (Better indicated by lower values)**												
2	randomised trials	no serious risk of bias	no serious inconsistency	no serious indirectness	no serious imprecision	none	100	100	-	MD 2.44 lower (3.32 to 1.55 lower)	⊕⊕⊕⊕ HIGH	IMPORTANT
**Symptom (Better indicated by lower values)**												
2	randomised trials	no serious risk of bias	no serious inconsistency	no serious indirectness	no serious imprecision	none	100	100	-	MD 2.31 lower (3.02 to 1.6 lower)	⊕⊕⊕⊕ HIGH	IMPORTANT
**MLHFQ (Better indicated by lower values)**												
6	randomised trials	serious[1]	no serious inconsistency	no serious indirectness	no serious imprecision	none	203	206	-	MD 7 lower (9.54 to 4.45 lower)	⊕⊕⊕O MODERATE	CRITICAL

[1] No explanation was provided

Author(s):
Date: 2023-11-15
Question: Subgroups analysis for cardiovascular disease

Settings:
Bibliography: . BDJ exercise for cardiovascular disease. Cochrane Database of Systematic Reviews [Year], Issue [Issue].

Quality assessment							No of patients		Effect		Quality	Importance
**No of studies**	**Design**	**Risk of bias**	**Inconsistency**	**Indirectness**	**Imprecision**	**Other considerations**	**Subgroups analysis**	**Control**	**Relative (95% CI)**	**Absolute**		
**LVEF (Better indicated by lower values)**												
15	randomised trials	serious[1]	no serious inconsistency	no serious indirectness	no serious imprecision	none	656	661	-	MD 4.25 higher (2.32 to 6.17 higher)	⊕⊕⊕O MODERATE	CRITICAL
**LVEF - Aged from 50 to 65 (Better indicated by lower values)**												
8	randomised trials	serious[1]	no serious inconsistency	no serious indirectness	no serious imprecision	none	350	354	-	MD 4.92 higher (1.09 to 8.74 higher)	⊕⊕⊕O MODERATE	
**LVEF - Aged above 65 (Better indicated by lower values)**												
7	randomised trials	serious[1]	no serious inconsistency	no serious indirectness	no serious imprecision	none	306	307	-	MD 3.59 higher (1.44 to 5.74 higher)	⊕⊕⊕O MODERATE	CRITICAL
**6-minute walking test (Better indicated by lower values)**												
15	randomised trials	serious[1]	no serious inconsistency	no serious indirectness	no serious imprecision	none	545	544	-	MD 42.95 higher (31.29 to 54.62 higher)	⊕⊕⊕O MODERATE	CRITICAL
**6-minute walking test - Aged from 50 to 65 (Better indicated by lower values)**												
7	randomised trials	serious[1]	no serious inconsistency	no serious indirectness	no serious imprecision	none	214	215	-	MD 41.67 higher (17.39 to 65.95 higher)	⊕⊕⊕O MODERATE	CRITICAL
**6-minute walking test - Aged above 65 (Better indicated by lower values)**												
8	randomised trials	no serious risk of bias	no serious inconsistency	no serious indirectness	no serious imprecision	none	331	329	-	MD 43.11 higher (31.94 to 54.29 higher)	⊕⊕⊕⊕ HIGH	CRITICAL
**NT-proBNP (Better indicated by lower values)**												
9	randomised trials	no serious risk of bias	no serious inconsistency	no serious indirectness	no serious imprecision	none	359	-	-	not pooled	⊕⊕⊕⊕ HIGH	CRITICAL
**NT-proBNP - Aged from 50 to 65 (Better indicated by lower values)**												
5	randomised trials	serious[1]	no serious inconsistency	no serious indirectness	no serious imprecision	none	184	-	-	not pooled	⊕⊕⊕O MODERATE	CRITICAL
**NT-proBNP - Aged above 65 (Better indicated by lower values)**												
4	randomised trials	no serious risk of bias	no serious inconsistency	no serious indirectness	no serious imprecision	none	175	-	-	not pooled	⊕⊕⊕⊕ HIGH	CRITICAL
**MLHFQ (Better indicated by lower values)**												
7	randomised trials	serious[1]	no serious inconsistency	no serious indirectness	no serious imprecision	none	247	253	-	MD 6.48 lower (8.92 to 4.03 lower)	⊕⊕⊕O MODERATE	CRITICAL
**MLHFQ - Aged from 50 to 65 (Better indicated by lower values)**												
5	randomised trials	serious[1]	no serious inconsistency	no serious indirectness	no serious imprecision	none	162	169	-	MD 6.38 lower (9.45 to 3.3 lower)	⊕⊕⊕O MODERATE	CRITICAL
**MLHFQ - Aged above 65 (Better indicated by lower values)**												
2	randomised trials	serious[1]	no serious inconsistency	no serious indirectness	no serious imprecision	none	85	84	-	MD 6.77 lower (12.69 to 0.85 lower)	⊕⊕⊕O MODERATE	CRITICAL

[1] No explanation was provided
